# A Multi-Country Comparison of Number Needed to Vaccinate for PCV20 and PCV15 in Infants

**DOI:** 10.3390/vaccines14020188

**Published:** 2026-02-18

**Authors:** Euan Dawson, Maria J. Tort, An Ta, Mark H. Rozenbaum

**Affiliations:** 1 Pfizer Inc., Tadworth KT20 7NS, UK; 2 Pfizer Inc., Collegeville, PA 19426, USA; 3 Cytel, Inc., London WC1H 9BB, UK; 4 Pfizer Inc., 2909 Capelle a/d Ijssel, The Netherlands

**Keywords:** vaccine-preventable diseases, number needed to vaccinate, pneumococcal conjugate vaccines, pediatric

## Abstract

**Background/Objectives:** Infant pneumococcal conjugate vaccines (PCV) have significantly reduced pneumococcal morbidity and mortality. Newer vaccines, 15-valent (PCV15) and 20-valent (PCV20), offer broader serotype coverage, potentially preventing more disease. This study estimated the number needed to vaccinate (NNV) to prevent one disease outcome for infant PCV20 and PCV15 programs versus 13-valent PCV (PCV13). Countries from Europe, the Asia-Pacific, and the Americas were included. **Methods:** A multi-cohort, population-based model estimated the cumulative NNVs for infant programs with PCV20 and PCV15 relative to PCV13 in 21 countries. Outcomes included overall pneumococcal case, hospitalization, and death. The ratio of PCV15 NNVs to PCV20 NNVs was calculated. Probabilistic sensitivity analysis (PSA) and scenario assessments tested results’ robustness. **Results:** Across 21 countries, the median of country-specific NNV estimates to prevent one pneumococcal case was 13 with PCV20 and 80 with PCV15. Median NNVs to prevent a hospitalization or death were 44 and 568 with PCV20 and 203 and 2203 with PCV15, respectively. PCV20 demonstrated lower NNVs than PCV15 across all countries and outcomes. Median NNV ratios for PCV15 versus PCV20 were 5.1 (case), 4.5 (hospitalization), and 4.2 (death). No clear geographic differences were observed. PSA and scenario analyses indicated stable results with minimal deviations. **Conclusions:** Infant immunization with PCV20 is associated with lower NNVs than PCV15. To achieve the same disease reduction as PCV20, over five times as many children would need to be vaccinated with PCV15. These findings suggest PCV20 may offer greater public health impact compared with PCV15 in infant immunization programs.

## 1. Introduction

Pneumococcal disease, caused by *Streptococcus pneumoniae* (*S. pneumoniae*), poses a significant public health concern due to its substantial morbidity and mortality [1]. Pneumococcal disease may manifest as invasive pneumococcal disease (IPD), including severe forms such as bacteremia and meningitis, or non-invasive pneumococcal disease, characterized by non-bacteremic pneumonia (NBP) and otitis media (OM) [2]. The burden is greatest among vulnerable populations such as children under the age of 2 years, adults aged 50 years and older, and individuals with chronic or immunocompromising conditions [2]. Globally, *S. pneumoniae* remains a leading cause of respiratory infections worldwide, accounting for 179,354 deaths and 15.8 million disability-adjusted life years among children in 2021 [3]. The burden is particularly pronounced in parts of Asia, where an estimated 53,240 child deaths were attributed to pneumococcal disease that same year [3]. Older adults are also significantly affected; in 2022, the incidence of IPD among those aged 65 years and older was 12.6 cases per 100,000 in Europe and 17.2 cases per 100,000 in the United States [4,5].

To reduce this burden, childhood immunization with pneumococcal conjugate vaccines (PCVs) has proven to be a highly effective public health intervention. The introduction of the infant 7-valent PCV (PCV7) in 2000 marked a major milestone in preventing vaccine-type pneumococcal disease. For example, the United States (US) experienced an approximate 95% reduction in IPD incidence among children under five years of age between 1998 and 2021 following widespread PCV use [6]. Beyond direct protection, infant vaccination confers substantial herd effects by reducing nasopharyngeal carriage of vaccine serotypes, thereby lowering transmission and disease incidence across all age groups [7]. PCV7 provides protection against seven serotypes of *S. pneumoniae* (serotypes 4, 6B, 9V, 14, 18C, 19F, and 23F), but the emergence of additional disease-causing serotypes prompted the development of higher-valency vaccines. The 13-valent PCV (PCV13; including 6 additional serotypes compared to PCV7: 1, 3, 5, 6A, 7F, and 19A), introduced in 2010, expanded protection and has since become the global standard of care (SoC). This vaccine is currently included in the national immunization programs (NIPs) of 134 countries [8]. Next-generation vaccines such as PCV15 (covering two additional serotypes compared to PCV13: 22F and 33F) and PCV20 (covering five more serotypes than PCV15: 8, 10A, 11A, 12F, and 15B) formulations have been developed and approved in several countries.

These higher-valent PCVs are currently at different stages of adoption globally. PCV15 has recently been introduced into some NIPs, such as in Lithuania, Iceland, and Hong Kong [9], while PCV20 has been implemented in countries such as Australia, Portugal, Israel, and Argentina [10,11,12,13,14,15]. Despite their expanded serotype coverage, these higher-valent PCVs have yet to be widely adopted into infant NIPs, indicating a need for additional evidence to support decision-making and guide resource allocation.

Cost-effectiveness analyses (CEAs) have traditionally been used to inform policy decisions by comparing the clinical and economic value of new vaccines. Recent CEAs have consistently demonstrated that pediatric use of PCV20 could substantially reduce pneumococcal disease burden compared with PCV13 and PCV15 [16]. While CEAs provide important insights, they often rely on country-specific data such as healthcare costs, limiting the comparability of results across regions. In this context, the number needed to vaccinate (NNV) has emerged as a valuable complementary metric. Analogous to the number needed to treat (NNT), NNV represents the average number of individuals who need to be vaccinated to prevent a single case of disease. As a standardized clinical outcome measure, NNV is particularly useful for comparing the potential impact of different vaccination programs across healthcare settings [17,18]. It provides an intuitive summary of vaccine effectiveness that is easily understood by healthcare providers and policymakers. NNV has been applied to evaluate various immunization programs, including those for COVID-19 [19,20,21], herpes zoster [22], and seasonal influenza [23]. However, research on NNVs for higher-valent PCVs is limited; only one study from the US compared PCV20 with PCV13, and it did not include PCV15 [24].

To fill this evidence gap, the current study aims to estimate the NNV for infant vaccination programs with PCV20 and PCV15, compared to PCV13, across multiple countries spanning Europe, Asia-Pacific, and the Americas.

## 2. Methods

This study estimated the NNVs for PCV20 and PCV15 across three primary outcomes including pneumococcal disease cases, hospitalizations, and deaths, which were then compared against PCV13. PCV13 was considered the SoC, given that it was implemented in the NIPs of the included countries at the time the study was conducted. PCV15 and PCV20 were not directly compared because neither had achieved widespread adoption at the time of writing, however the ratios of NNV for PCV15 to PCV20 were calculated and used as a tool for comparison.

Countries were included based on availability of published modeling studies with sufficient technical detail to allow harmonized calculations of NNV outcomes available at the time of the analysis [25,26,27,28,29,30,31,32,33,34,35,36,37,38,39,40,41,42,43,44,45]. Only publicly available models with sufficient methodological transparency were included; unpublished or proprietary models were excluded. A total of 21 countries were included, representing the Americas (Argentina, Canada, Chile, Mexico, and the US), Europe (Belgium, France, Germany, Greece, Italy, Portugal, Romania, Slovakia, Spain, and Sweden) and the Asia-Pacific region (Australia, Japan, Malaysia, Singapore, South Korea, and Taiwan), each with a different healthcare system, population structure, and pneumococcal disease trend, but all with PCV13 as a common SoC. At the time of this analysis, these 21 countries were the only publicly available records—drawn from peer-reviewed publications and conference proceedings—for which all required data were available to run the model.

Data from all models were independently reviewed and extracted to adapt the NNV models for all included countries by one modeler, and all models were then independently reviewed by a second modeler using a standardized template to ensure consistency in the adaptations, and to minimize risk of transcription error. Any disagreements were resolved through discussion with a third reviewer. A full list of local inputs and their sources is provided in Supplementary Materials (Table S2).

### 2.1. Model Overview

A previously published model with a 25-year time horizon was used to calculate the cumulative NNV for each country [24]. The model used a population-based multiple-cohort Markov structure to estimate the clinical outcomes of different pediatric PCV programs over the time horizon [24]. In the model, annual birth cohorts of children aged <2 years old were routinely vaccinated over 25 years with either PCV13, PCV15, or PCV20, depending on the comparison. The entire population stratified by age (see Model inputs—Population) was included in the model to capture the direct effects on the vaccinated population and the indirect effects on the unvaccinated population. The clinical outcomes were cases of IPD, inpatient and outpatient NBP, and OM, as well as deaths that were prevented due to each vaccination strategy.

The clinical outcomes generated by the model were utilized to inform the NNV calculation. The approach used in the analysis was based on a methodology in Brisson et al. [46], where

$$NNV=\frac{Number\;vaccinated}{Cases\;prevented\;over\;time\;horizon\;}$$

Contrary to traditional NNV calculations, this model-based approach incorporated the cumulative impact of vaccination over time, which enabled the inclusion of both direct and indirect effects of pediatric vaccination [47]. This approach also means comparisons can be made between the vaccine of interest (PCV20 or PCV15) and the SoC (PCV13) [24]. The analysis extracts annual birth cohort data from the original models and assumes that the birth cohort in year 10 is representative of subsequent cohorts and is held constant through year 25. Vaccine uptake was assumed to remain stable over the time horizon, based on country-specific coverage data, and applied consistently across vaccination strategies.

### 2.2. Model Inputs

All input data required to estimate the NNV were extracted from published studies and included information on the vaccine, the epidemiology, and the population [25,26,27,28,29,30,31,32,33,34,35,36,37,38,39,40,41,42,43,44,45]. While model parameters relating to vaccine effectiveness and duration of protection were mostly consistent across countries, parameters related to population structure, epidemiology, and vaccine uptake were country-specific to account for local heterogeneity. Parameters and data sources for all 21 countries can be found in the original publications with key local inputs summarized in the Supplementary Material (Table S1) [25,26,27,28,29,30,31,32,33,34,35,36,37,38,39,40,41,42,43,44,45].

An overview of the key base-case parameters is provided in Table 1.

### 2.3. Population

The model included the entire population in each local setting and was stratified according to age groups (<12 months, 12–23 months, 24–35 months, 36–49 months, 5–17 years, 18–49 years, 50–64 years, and 65+ years). Estimates of the population and projected annual birth cohort sizes were obtained from the published country-specific sources.

### 2.4. Disease Incidence Rates

Age-specific incidence rates per 100,000 individuals were used for each disease manifestation. In line with the studies from which the data were extracted, IPD incidences were assumed to be classified as either meningitis or bacteremia, with the overall IPD incidence calculated as the sum of these two manifestations. Similarly, non-invasive diseases including pneumonia and OM were defined in a broader category as all-cause disease, encompassing cases from any etiology rather than those caused by *S. pneumoniae* only. This approach reflects the limited availability of etiology-specific data and remains consistent with the modeling approach in economic evaluations of PCVs. NBP rates were separated by inpatient or outpatient setting. OM rates were not classified by setting and represented all cases of OM in the pediatric population. It is noted that while certain countries differentiated OM into complex and simple forms, this study aggregated outcomes for all OM cases to facilitate consistent and comparable reporting across regions. OM is infrequent in the adult population, so no cases were conservatively assumed to occur among adults. Similarly, no sequelae or long-term consequences were considered in the model given low prevalence as well as the absence of robust local data. This exclusion is expected to have a limited impact on the results and is likely conservative. In a previous US cost-effectiveness analysis, which used a similar modeling framework, sequelae accounted for approximately 4% of total costs and less than 5% of total QALY losses [27].

### 2.5. Case Fatality Ratio and Mortality Rates

The model used general population mortality rates to estimate background mortality (deaths from all causes), while age- and disease-specific case-fatality rates (CFRs), informed by local data, were applied to estimate pneumococcal disease-related deaths. Outpatient NBP and OM cases were not assumed to be associated with elevated mortality.

### 2.6. Serotype Coverage

The serotype distribution for each PCV was informed by local IPD surveillance data and stratified by age. In the absence of serotype-specific data for non-invasive disease, the serotype coverage was assumed to be equivalent to the IPD. This assumption was uniform across all studies analyzed.

### 2.7. Vaccine Uptake

The vaccine uptake was specific to local coverage data. This analysis preserved the schedule used in the original country-specific evaluations to ensure methodological consistency. PCV13, PCV15, and PCV20 were administered according to local immunization schedules, either 2 + 1 or 3 + 1, depending on the country.

### 2.8. Vaccine Effectiveness

Widespread use of infant PCVs has led to reductions in pneumococcal disease in both the vaccinated and the unvaccinated population [8,48,49,50]. Therefore, the model accounted for both the direct effects and indirect effects of infant vaccination.

### 2.9. Direct Effectiveness

To enable consistent cross-country comparison, and align with the local CEAs, we preserved the assumption that vaccine effectiveness against disease for PCV15 and PCV20 was equivalent to that of PCV13. Due to the lack of vaccine effectiveness studies and real-world data for PCV15 and PCV20 in infants at the time of the study, direct vaccine effectiveness estimates against IPD were sourced from vaccine effectiveness and real-world studies of PCV13 [51]. This approach followed early post-licensure modeling practice and ensured consistency across countries. Estimates for the majority of countries were obtained from a recent European multicenter study assessing PCV13 under both 2 + 1 and 3 + 1 schedules [51], while US-specific data were used for the US, Japan, Canada, and South Korea, where all PCVs are administered under a 3 + 1 schedule [52]. Direct effectiveness estimates against non-invasive diseases (pneumonia and OM) were based on efficacy data from clinical trials reporting on PCV7 [53,54,55] and were consistent across all countries.

The duration of protection for all PCVs was assumed to be 15 years, with the direct effect gradually waning over time. The full vaccine effectiveness was assumed to be maintained for the first four years after the booster dose, after which it was assumed to decrease by 10% annually from year 6 to year 15, beyond which no residual vaccine effectiveness was considered [51].

#### Indirect Effects

The estimation of indirect effects incorporated the rate of accrual, reflecting the progressive build-up of indirect protection over time until a steady state was reached (i.e., after this no additional protection is expected) and the maximum reduction in pneumococcal disease incidence once the steady state was achieved.

Country-specific (local) data were used to inform maximum reduction in IPD incidence from indirect effect, and details can be found in Table S1 as well as the original study publications from which these data were extracted [56,57,58,59,60,61]. By contrast, countries including Australia, Belgium, Malaysia, The Netherlands, Portugal, Spain and Sweden were informed through an observational study of PCV13 on the UK [62]. For non-invasive disease (pneumonia and OM), most countries adopted the same estimates from PCV13 impact studies [58,59,60,61,62,63,64,65], except for Sweden and the US, which used country-specific estimates based on real-world evidence [66,67,68,69].

The accrual rate inputs also varied across countries. Most settings followed the same assumption based on Ladhani et al. [62] and Perdrizet et al. [8], whereas distinct accrual patterns were used for the US, Canada [63], France [58,59], Taiwan [61], Argentina [56], Peru [56] and Chile [56] based on country-specific evidence and epidemiological dynamics [25,26,27,28,29,30,31,32,33,34,35,36,37,38,39,40,41,42,43,44,45].

### 2.10. Primary Analyses

Pairwise comparisons of PCV20 versus PCV13 and PCV15 versus PCV13 were conducted for each country in the primary analyses. The modeled outcomes included disease cases of IPD, inpatient and outpatient NBP, OM, and disease-related deaths prevented under each vaccination strategy. The NNVs for each outcome were computed following the approach described in Brisson et al. [46]. Regional (Americas, Europe, Asia-Pacific) and global results were obtained by combining country-level outcomes. When aggregating across countries, unweighted medians, interquartile ranges (IQR), and confidence intervals were reported to enable comparisons and inference. Unweighted medians rather than population-weighted estimates were chosen to reflect a typical country-level experience and to avoid disproportionate influence of a small number of large population countries on aggregated results.

PCV20 and PCV15 were each compared with PCV13 for every country. We estimated how many pneumococcal disease cases, hospitalizations, and deaths could be prevented and calculated how many children would need to be vaccinated to prevent each outcome. NNV outcomes were estimated separately for each country, from which a ratio for the NNV of PCV15 to PCV20 was calculated. Results were analyzed by country and then summarized at regional and global levels using medians and ranges to enable comparison across settings.

### 2.11. Sensitivity Analyses

#### Scenario Analyses

Scenario analyses were conducted to assess the impact of uncertainty in key parameters on the model outcomes. Firstly, since no real-world vaccine effectiveness data are yet available for infant vaccination with PCV20 and PCV15, the direct vaccine effectiveness for the serotypes included into PCV15 and PCV20 was assumed to be equivalent to PCV13 in the primary analyses. To test this assumption, a more conservative scenario incorporating a 20% reduction in PCV20 and PCV15 vaccine effectiveness was evaluated. Secondly, the serotype distribution used in the models is subject to uncertainty because the data are often drawn from small samples of IPD cases. Additionally, the IPD distribution may not be representative of the serotype distribution of non-invasive disease like NBP and OM, for which the distribution is usually unknown due to detection limitations. Therefore, sensitivity assessments were performed by varying the serotype distribution of PCV20 and PCV15 by ± 25%. Thirdly, to evaluate the assumption on duration of protection, a 10-year duration of protection for all PCVs, as opposed to the 15-year time horizon in the primary analyses, was adopted. Finally, since data for indirect effects of PCV15 and PCV20 were extrapolated from PCV13 experience, potential variation in higher-valent formulations was tested in a conservative scenario, reducing herd effects by 25%. These scenario analyses were performed for a representative sample of countries, which included Italy, Germany, the US, Canada, Japan, and Australia. These countries were selected to be broadly representative of the geographic regions and to capture heterogeneity in the model parameters.

### 2.12. Probabilistic Sensitivity Analysis

A PSA was conducted for all countries with 1000 iterations to test the joint uncertainty of the input parameters. Probability distributions were assigned to all relevant parameters based on Briggs et al., such as a beta distribution applied to disease incidence and utility parameters, while a log-normal distribution was applied to mortality rates [70].

## 3. Results

### 3.1. Base-Case Analysis

**Figure 1:**
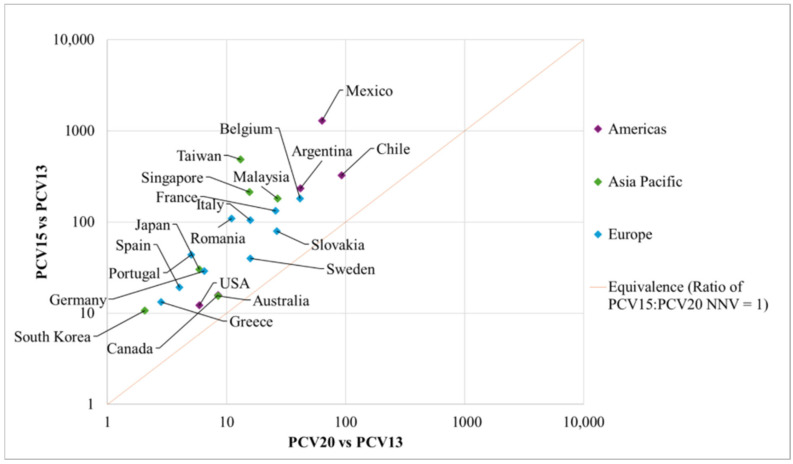
NNVs by country and region, per disease case prevented for PCV20 vs. PCV13 and PCV15 vs. PCV13. Abbreviations: NNV, number needed to vaccinate; PCV13, 13-valent pneumococcal conjugate vaccine; PCV15, 15-valent pneumococcal conjugate vaccine; PCV20, 20-valent pneumococcal conjugate vaccine.

**Figure 2:**
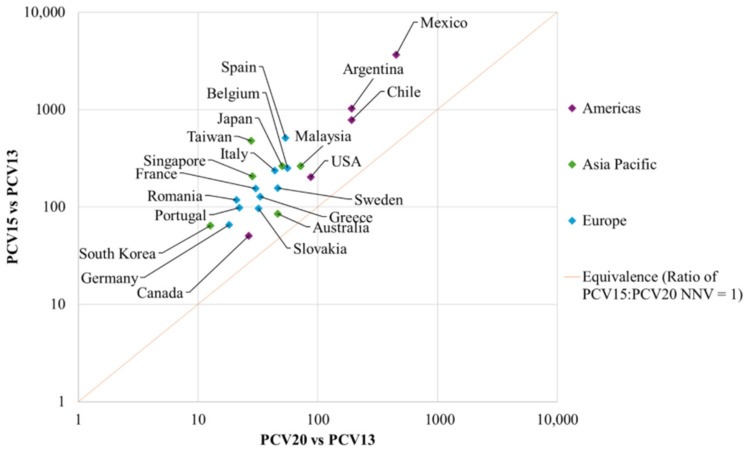
NNVs by country and region, per hospitalization prevented for PCV20 vs. PCV13 and PCV15 vs. PCV13. Abbreviations: NNV, number needed to vaccinate; PCV13, 13-valent pneumococcal conjugate vaccine; PCV15, 15-valent pneumococcal conjugate vaccine; PCV20, 20-valent pneumococcal conjugate vaccine.

**Figure 3:**
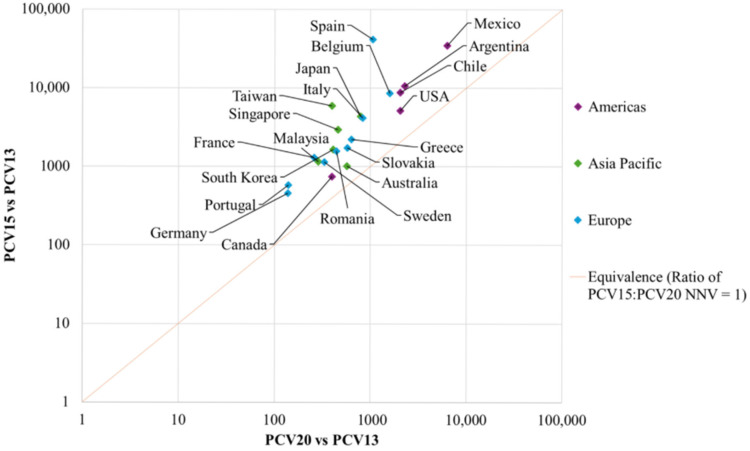
NNVs by country and region, per death prevented for PCV20 vs. PCV13 and PCV15 vs. PCV13. Abbreviations: NNV, number needed to vaccinate; PCV13, 13-valent pneumococcal conjugate vaccine; PCV15, 15-valent pneumococcal conjugate vaccine; PCV20, 20-valent pneumococcal conjugate vaccine.

The NNVs for PCV20 and PCV15 to prevent a disease case, hospitalization, and death versus PCV13 in each country, and the median NNVs according to region are presented in Table 2, and Figure 1, Figure 2 and Figure 3, respectively. The ratios of NNVs for PCV15 and PCV20 are presented in Table 3.

Additional event-specific data for IPD, inpatient and outpatient NBP, and OM can be found in Table S1.

### 3.2. Overall PD Case

Across all countries, the median NNVs to prevent a case of pneumococcal disease were 13 for PCV20 and 80 for PCV15 compared against PCV13 (Table 2). The median ratios of PCV15 NNVs to PCV20 NNVs for one case of pneumococcal disease were 5.1 across all countries. Regionally, the median NNVs for PCV20 were 13 in Europe, 11 in Asia, and 42 in the Americas, while the figures for PCV15 were 62 in Europe, 102 in Asia, and 235 in the Americas. When indirectly compared by the median NNV ratios, the ratios of PCV15 to PCV20 were 4.7 in Europe, 5.8 in Asia, and 3.5 in the Americas.

Individual country NNVs for a case of pneumococcal disease for PCV20 ranged from 2 in South Korea to 92 in Chile, while the PCV15 NNVs ranged from 11 in South Korea to 1289 in Mexico (Table 2). Ratios in European countries ranged from 2.5 in Sweden to 9.9 in Romania, in Asia-Pacific countries from 1.8 in Australia to 35.3 in Taiwan, and in the Americas from 1.9 in Canada to 20.4 in Mexico. Figure 1 presents the indirect comparison of PCV20 NNVs and PCV15 NNVs, graphically demonstrating where the country estimates lay relative to the line of equivalence. All countries fell above the line of equivalence, indicating that the NNVs with PCV20 were lower than the NNV with PCV15 for a case of pneumococcal disease (Figure 1).

### 3.3. One Hospitalization

Across all countries, the median NNVs to prevent one hospitalization related to pneumococcal disease were 44 for PCV20 and 203 for PCV15. The median ratios of PCV15 NNVs to PCV20 NNVs showed that for one hospitalization, the ratio was 4.5 in the global setting. Regionally, the median NNVs for PCV20 were 33 in Europe, 37 in Asia, and 191 in the Americas, while the median NNVs for PCV15 were 142 in Europe, 235 in Asia, and 786 in the Americas. When indirectly compared to the median ratios of PCV15 to PCV20, the estimates were 4.5 in Europe, 5.2 in Asia, and 4.1 in the Americas.

Individual country NNVs for PCV20 ranged from 13 in South Korea to 451 in Mexico, while PCV15 NNVs ranged from 51 in Canada to 3666 in Mexico (Table 2). Median ratios in European countries ranged from 3.0 in Slovakia to 9.6 in Spain, in Asian countries from 1.8 in Australia to 17.3 in Taiwan, and in the Americas from 1.9 in Canada to 8.1 in Mexico. Figure 2 demonstrates that all countries fell above the line of equivalence, indicating that the NNVs with PCV20 were lower than the NNVs with PCV15 for a hospitalization related to pneumococcal disease.

### 3.4. One Death

Across all countries, the median NNVs to prevent a death related to pneumococcal disease were 568 for PCV20 and 2203 for PCV15. The median ratios of PCV15 NNVs to PCV20 NNVs showed that for one death, the ratio was 4.2 in the global setting. Regionally, the median NNVs for PCV20 were 509 in Europe, 435 in Asia, and 2053 in the Americas, while the median NNVs for PCV15 were 1651 in Europe, 2297 in Asia, and 8781 in the Americas. The median ratios of PCV15 to PCV20 were 3.9 in Europe, 4.8 in Asia, and 4.3 in the Americas.

Individual country NNVs for PCV20 ranged from 138 in Germany to 6327 in Mexico, and PCV15 NNVs ranged from 458 in Germany to 41,593 in Spain (Table 2). Median ratios in European countries ranged from 3.0 in Slovakia to 39.5 in Spain, in Asian countries from 1.8 in Australia to 15.0 in Taiwan, and in the Americas from 1.9 in Canada to 5.5 in Mexico. Figure 3 demonstrates that all countries lay above the line of equivalence, indicating that the NNVs with PCV20 were lower than the NNV with PCV15 for a death related to pneumococcal disease.

### 3.5. Scenario Analyses

The results of the four scenarios for each of the six countries are presented in Table 4 and Table 5.

When the duration of vaccine protection was reduced from 15 years to 10 years, the NNVs for PCV20 and PCV15 in all countries were marginally greater than the primary analysis estimates for each outcome. Similarly, when direct vaccine effectiveness was reduced by 20%, there was marginally greater NNVs than the results in the primary analysis for each outcome. Reducing indirect effects by 25% led to a notable increase in the NNVs for PCV20 and PCV15 for each outcome compared to the primary analysis estimates. Likewise, the impact of increasing serotype coverage of each vaccine by 25% caused the NNVs to decrease significantly for all outcomes, while a 25% decrease in the serotype coverage rates resulted in significant increase in NNVs for all outcomes. The ratios of PCV15 to PCV20 NNVs generally remained the same regardless of the parameters that were varied in the scenario analyses.

### 3.6. Probabilistic Sensitivity Analysis

PSA results are presented by country and by region in Table 6. In all countries, the overall probabilistic estimates were closely aligned with the primary analyses estimates.

## 4. Discussion

This study demonstrated that infant immunization with PCV20 was associated with substantially lower NNVs compared with PCV15 in preventing pneumococcal disease cases, hospitalizations, and deaths across 21 predominantly high-income countries in Europe, Asia-Pacific, and the Americas. When compared to infant vaccination with PCV13, the median ratios of PCV15 to PCV20 indicated that around 4 to 5 times more infants would need to be vaccinated with PCV15 to achieve the same health impact as PCV20.

Regional analyses showed that the median NNVs in Europe and Asia-Pacific were similar, while the median NNVs in the Americas were more than three times higher. For example, the median NNVs to prevent a case of pneumococcal disease with PCV20 was 42 in the Americas compared to 13 in Europe and 11 in Asia-Pacific. This variation was largely driven by higher NNV observed in Mexico, Argentina, and Chile. This result was likely driven by delayed introduction of PCV7 and PCV13 infant programs in these countries compared to many European and Asia-Pacific settings. Consequently, a larger proportion of the remaining pneumococcal disease burden is attributable to PCV13 serotypes, leading to a reduced incremental benefit to serotypes unique to PCV20. No other consistent regional trends were identified.

At the country level, PCV20 consistently demonstrated lower NNVs than PCV15 across all outcomes as reflected by ratios of PCV15 to PCV20 being greater than one in every setting. The lowest ratios were observed in Canada, Australia, and the US (at around 2), indicating that even with a small ratio, twice as many children would need to be vaccinated with PCV15 to achieve the same population-level impact as PCV20. Conversely, the highest ratios were observed in Mexico, Taiwan and Spain. In countries where there were higher NNV ratios, the difference between vaccine serotype coverage for PCV15 and PCV13 was minimal (<5%). PCV15 only contains two incremental serotypes compared to PCV13; therefore, in many settings, the additional health benefit is relatively minor. Consequently, in these countries, significantly more infants would need to be vaccinated with PCV15 to prevent a disease outcome relative to use with PCV13. Since PCV20 contains seven additional serotypes compared to PCV13, there is generally considerable additional health benefit because PCV20 protects against several prevalent serotypes.

This study adopted the same methodological framework, assumptions and input data used in previous CEA studies of PCVs, ensuring consistency in structural characteristics and key drivers of the model outcomes. Scenario analyses provided valuable insights into the robustness of the primary analyses results, the sensitivity of model outcomes and key parameters. In particular, we assessed four key parameters including vaccine direct effectiveness, serotype coverage, duration of protection, and the indirect effect, among which the maximum reduction in disease incidence attributable to indirect effects emerged as the most influential variable, having the greatest impact on the NNVs across all disease outcomes. This finding aligns with previous studies highlighting the importance of herd immunity in determining the overall public health impact of PCVs, particularly in pediatric immunization. Notably, all scenarios and PSAs yielded results consistent with the primary analysis, with only modest deviations observed. These findings provided strong support for the validity and stability of the model and reinforce the robust comparative advantage of PCV20 over PCV15, even under conservative or alternative parameter assumptions.

This is the first study to estimate the NNVs for higher-valent pediatric PCVs across a diverse set of global settings. The findings are consistent with the limited literature on this topic such as recent US- and German-based modeling studies, as well a systematic review. For example, a US-based study reported an NNV of six to prevent one case of pneumococcal disease, with event-specific NNVs for IPD, inpatient NBP, outpatient NBP, and OM of 854, 106, 25 and 9, respectively [24]. Similarly, Kuhlmann et al. used a dynamic transmission model to compare the NNV for both PCV15 and PCV20 against PCV13 in Germany, reporting results broadly consistent with our findings, such as an NNV of 23 to prevent one hospitalization, and 177 to prevent a death (versus 17 and 138 in our study, respectively) [71]. Additionally, a recent systematic review concluded that switching from PCV13 to PCV20 would be more beneficial in terms of direct cost reduction and would yield a bigger gain in QALY compared to switching from PCV13 to PCV15 [16].

A major strength of the current study lied in the inclusion of both direct and indirect effects of infant vaccination, enabling a more comprehensive assessment of the public health impact of PCVs. The importance of accounting for indirect effects in NNV calculations was highlighted in a recent study, which emphasized that cumulative direct and indirect effects should be included to capture the full public health impact on the whole population [24]. Nevertheless, the assumptions made in the model for indirect effects are also subject to limitations. There is uncertainty around future indirect effects due to PCV15 and PCV20 infant vaccination. To remain conservative, the model assumed that a proportion of the adult population would not receive any benefit from the indirect effect of pediatric vaccination.

There are some limitations that should be discussed. Firstly, although pneumococcal carriage and transmission are a precursor to pneumococcal disease, the model used a static approach [16,72,73,74,75], which might have overestimated the advantage of PCV20. The recent literature from dynamic transmission modeling studies comparing the impact of PCV15 and PCV20 provides additional context for the interpretation of our findings. A study conducted by the UK Health Security Agency suggested that PCV15 may increase the overall pneumococcal disease burden compared with PCV13, whereas PCV20 was estimated to produce substantial reductions in disease incidence due to the higher invasiveness of its unique serotypes to the non-vaccine serotypes expected to replace them [76]. Similar results were reported from dynamic transmission models in the US, Israel, Malawi, and South Africa, indicating that PCV15 would offer only limited additional benefit over PCV13 [77,78]. Overall, these findings indicated that our estimates may overstate the impact of PCV15 and therefore conservatively represent the relative advantage of PCV20 in reducing pneumococcal disease burden.

Secondly, our static model did not consider serotype replacement, which may occur during the time horizon as serotype epidemiology changes in response to vaccine uptake. Vaccine serotypes may become less prevalent as a proportion of overall disease, altering the NNV outcome over time. However, the dynamics of serotype replacement are not well understood. To explore the impact of serotype replacement, a 25% reduction in the serotype coverage of PCV20 and PCV15 was modeled for six countries in the scenario analyses, which demonstrated that the NNV of PCV20 would remain lower than the NNV of PCV15. Furthermore, the observed indirect effects for PCV7 and PCV13 were extrapolated to higher-valent PCVs which is a common methodological practice in cost-effectiveness analyses for new PCVs due to the absence of real-world data at the time of introduction. However, this approach could result in either an over or underestimation of the herd effects.

Thirdly, due to lack of serotype coverage data for non-invasive diseases, we assumed the same distribution between non-invasive serotypes and invasive serotypes. In reality, non-invasive disease syndromes may exhibit distinct serotype patterns. This approach has, however, been used in previous cost-effectiveness models as data on non-invasive serotypes are scarce. In addition, because pneumonia and otitis media were modeled as all-cause outcomes, the absolute number of vaccine-preventable events may be over- or under-estimated since the underlying proportion of the disease due to *S. pneumoniae* is not well characterized. Although this methodology is commonly employed in economic evaluations of PCVs due to the challenges in pathogen detection for non-invasive syndromes, it may provide a slight advantage to PCV20 because of its broader serotype coverage compared to PCV13 and PCV15. Nevertheless, given that the same assumption was uniformly applied across all three vaccines—PCV13, PCV15, and PCV20—the relative comparisons and NNV ratios are expected to remain reliable and are unlikely to be materially affected by this approach.

Moreover, equivalent vaccine effectiveness for PCV13-shared serotypes was assumed across vaccines. Although this assumption was commonly used in prior modeling studies and widely accepted, it does not capture potential immunogenicity differences observed in some trials. Higher valent vaccines may have lower immunogenicity than lower-valent ones, but this does not mean they are less effective. For example, an indirect study comparing PCV10 and PCV20 found their immunogenicity to be similar, indicating comparable effectiveness for the ten shared serotypes, with PCV10 being highly effective [79]. The emergence of real-world vaccine effectiveness data for PCV15 and PCV20 in infants would allow future analyses to instead use vaccine-specific effectiveness which could produce a more precise estimate of NNV. Additionally, although a harmonized analytic framework was applied, underlying differences in the structure and assumptions of the original country-specific models may influence absolute NNV estimates. Furthermore, the included countries in this study were predominantly high-income settings, reflecting where the most developed pneumococcal modeling infrastructure, surveillance systems, and publicly available evaluations and evidence in PCVs are from, rather than a selective sampling strategy. As a result, the study should not be interpreted as a globally representative sample and may have greater applicability for countries in high-income contexts. However, the sets of included countries spread across several geographic regions and diverse epidemiological profiles, enabling the examination of the consistency of comparative NNV outcomes across varied contexts.

Finally, while NNV is a useful metric enabling a measure of vaccine impact and providing evidence aiding decision-making, policymakers consider a broader range of factors beyond clinical efficacy. These include vaccine price, cost-effectiveness, supply constraints, budget impact, programmatic feasibility, co-administration with other vaccines and alignment with national priorities. The CDC’s Evidence to Recommendation (EtR) framework, for example, systematically evaluates such factors by assessing the quality of evidence, balance of benefits and harms, resource use, acceptability, and feasibility of implementation [80,81].

## 5. Conclusions

Infant immunization with PCV20 was consistently associated with substantially lower NNVs compared with PCV15 in preventing pneumococcal disease cases, hospitalizations, and deaths across 21 countries in Europe, Asia-Pacific, and the Americas. On average, to achieve the same reduction in disease burden as provided by PCV20, more than five times as many children would need to be vaccinated with PCV15.

## Figures and Tables

**Table 1:** Summary of base-case settings.

Category	Setting
Time horizon	25 years
Cycle length	One year
Vaccine schedules	2 + 1 or 3 + 1, depending on local recommendation and vaccine labels
Effects included	Direct and indirect effects included
Vaccine uptake	Country-specific estimates were used which were based on PCV13 coverage and assumed to be same for PCV15/PCV20
Vaccine waning	Assumed same for PCV13/PCV15/PCV20 No reduction in the first 4 years following the booster dose Waning started from year 6 at an annual 10% reduction up to year 15 (i.e., maximum duration of protection) From year 16 onward vaccine effectiveness assumed at 0%
Catch-up program	Not included in primary analyses
Population	Vaccinated cohort aged <2 years and unvaccinated cohort aged ≥2 years
Outcomes	NNV per case avoided NNV per hospitalization avoided NNV per death avoided Ratio of NNV (PCV20:PCV15)

Abbreviations: NNV, number needed to vaccinate; PCV13, 13-valent pneumococcal conjugate vaccine; PCV15, 15-valent pneumococcal conjugate vaccine; PCV20, 20-valent pneumococcal conjugate vaccine.

**Table 2:** Number needed to vaccinate to prevent one outcome, for each country and region included for PCV13 vs. PCV20 and PCV13 vs. PCV15.

	Case: PCV13 vs.		Hospitalization: PCV13 vs.		Death: PCV13 vs.	
Country	PCV20	PCV15	PCV20	PCV15	PCV20	PCV15
Argentina	42	235	191	1023	2269	10,581
Canada	8	16	26	51	398	747
Chile	92	328	192	786	2053	8781
Mexico	63	1289	451	3666	6327	34,587
US	6	12	87	203	2046	5128
Americas median (IQR)	42 (8–63)	235 (16–328)	191 (87–192)	786 (203–1023)	2053 (2046–2269)	8781 (5128–10,581)
Belgium	42	180	56	251	1592	8609
France	26	133	30	155	260	1307
Germany	7	29	18	65	138	458
Greece	3	13	33	128	636	2203
Italy	16	105	44	237	834	4162
Portugal	5	44	22	98	139	579
Romania	11	109	21	118	443	1573
Slovakia	26	80	32	97	576	1729
Spain	4	19	53	513	1054	41,593
Sweden	16	40	46	156	332	1144
Europe median (IQR)	13 (5–23)	62 (32–108)	33 (24–46)	142 (103–217)	509 (278–784)	1651 (1185–3672)
Australia	8	15	46	85	568	1018
Japan	6	30	50	263	789	4351
Malaysia	27	173	72	262	286	1149
Singapore	16	205	28	207	459	2941
South Korea	2	11	13	64	411	1654
Taiwan	13	465	28	480	397	5957
Asia-Pacific median (IQR)	11 (7–15)	102 (19–205)	37 (28–49)	235 (115–263)	435 (401–541)	2297 (1275–3998)
Overall median (IQR)	13 (6–26)	80 (19–181)	44 (28–56)	203 (98–263)	568 (397–1054)	2203 (1149–5957)

Abbreviations: IQR, interquartile range; PCV13, 13-valent pneumococcal conjugate vaccine; PCV15, 15-valent pneumococcal conjugate vaccine; PCV20, 20-valent pneumococcal conjugate vaccine; US, United States.

**Table 3:** Ratio of NNV for PCV15 vs. PCV20 by country and outcome, with overall median and IQR.

Country	Case	Hospitalization	Death
Argentina	5.6	5.3	4.7
Canada	1.9	1.9	1.9
Chile	3.5	4.1	4.3
Mexico	20.4	8.1	5.5
US	2.1	2.3	2.5
America’s median	3.5	4.1	4.3
Belgium	4.3	4.5	5.4
France	5.1	5.1	5.0
Germany	4.5	3.6	3.3
Greece	4.7	3.9	3.5
Italy	6.6	5.4	5.0
Portugal	8.6	4.4	4.2
Romania	9.9	5.7	3.6
Slovakia	3.0	3.0	3.0
Spain	4.8	9.6	39.5
Sweden	2.5	3.4	3.4
Europe median	4.7	4.5	3.9
Australia	1.8	1.8	1.8
Japan	5.1	5.2	5.5
Malaysia	6.4	3.7	4.0
Singapore	13.1	7.3	6.4
South Korea	5.2	5.1	4.0
Taiwan	35.3	17.3	15.0
Asia-Pacific median	5.8	5.2	4.8
Overall median (IQR)	5.1 (3.5–6.7)	4.5 (3.6–5.4)	4.2 (3.4–5.4)

Abbreviations: IQR, interquartile range; NNV, number needed to vaccinate; PCV15, 15-valent pneumococcal conjugate vaccine; PCV20, 20-valent pneumococcal conjugate vaccine. Note: NNV ratios are derived from an indirect comparison of PCV20 vs. PCV13 and PCV15 vs. PCV13.

**Table 4:** Scenario analysis of PCV20 NNV to prevent each outcome by country.

PCV20	Scenario 1			Scenario 2 (a,b)			Scenario 3			Scenario 4		
Country	Case	Hospitalization	Death	Case	Hospitalization	Death	Case	Hospitalization	Death	Case	Hospitalization	Death
Australia	8.8	46.7	568.7	6.8–11.3	36.9–61.4	454.5–757.8	8.5	46.4	568.4	10.6	59.6	756.1
Japan	6.1	51.8	789.3	4.7–7.9	40.3–67.1	631.1–1052.1	6.0	50.5	789.1	7.5	62.8	1050.3
US	6.2	88.8	2051.8	4.8–7.9	71.1–113.6	1702.1–2563.7	6.1	88.4	2053.6	7.3	112.5	2702.7
Canada	8.7	26.5	398.6	6.8–11.3	21.2–35.3	318.7–531.4	8.5	26.5	398.6	10.8	35.0	530.9
Italy	15.9	43.9	834.5	12.7–21.1	35.1–57.6	667.3–1109.9	15.9	43.8	834.3	21.0	57.5	1109.7
Germany	6.6	18.3	138.2	5.3–8.4	14.6–24.0	110.8–184.8	6.5	18.2	138.2	8.3	23.7	184.2

Scenario 1: Vaccine effectiveness reduced by 20%, Scenario 2a: Serotype coverage increased by 25%, Scenario 2b: Serotype coverage decreased by 25%, Scenario 3: Duration of protection reduced to 10 years, Scenario 4: Herd effects reduced by 25%.

**Table 5:** Scenario analysis of PCV15 NNV to prevent each outcome by country.

PCV15	Scenario 1			Scenario 2 (a,b)			Scenario 3			Scenario 4		
Country	Case	Hospitalization	Death	Case	Hospitalization	Death	Case	Hospitalization	Death	Case	Hospitalization	Death
Australia	16.0	86.0	1018.7	12.4–20.7	67.9–113.3	814.2–1357.3	15.7	85.8	1018.2	19.3	110.0	1354.3
Japan	31.3	271.9	4353.3	24.3–40.4	210.7–351.1	3480.8–5801.5	30.4	265.0	4352.0	37.9	327.0	5790.1
US	13.0	207.1	5150.7	9.8–16.4	162.3–270.6	4102.4–6837.5	12.6	205.5	5152.2	15.0	259.0	6755.3
Canada	16.2	50.8	747.3	12.6–21.1	40.5–67.5	597.7–996.4	15.9	50.7	747.5	20.1	66.8	995.3
Italy	106.0	241.5	4173.5	84.0–140.4	190.6–313.5	3331.9–5542.5	105.7	239.9	4173.2	137.6	314.9	5548.0
Germany	29.6	65.6	458.3	23.7–37.9	52.6–86.7	367.7–612.9	29.5	65.7	458.5	37.5	86.1	611.0

Scenario 1: Vaccine effectiveness reduced by 20%, Scenario 2a: Serotype coverage increased by 25%, Scenario 2b: Serotype coverage decreased by 25%, Scenario 3: Duration of protection reduced to 10 years, Scenario 4: Herd effects reduced by 25%.

**Table 6:** Probabilistic central estimates for mean NNV (95% confidence interval) to prevent each outcome by country based on 1000 runs (PCV13 vs. PCV20 and PCV13 vs. PCV15).

	Cases: PCV13 vs.		Hospitalizations: PCV13 vs.		Deaths: PCV13 vs.	
Country	PCV20	PCV15	PCV20	PCV15	PCV20	PCV15
Argentina	42 (39–45)	236 (221–251)	192 (178–206)	1026 (942–1114)	2279 (2074–2513)	10,617 (9482–11,831)
Canada	8 (8–9)	16 (15–17)	27 (13–31)	51 (43–59)	401 (338–470)	752 (631–882)
Chile	93 (87–98)	328 (309–349)	192 (177–208)	789 (727–853)	2060 (1847–2271)	8825 (7895–9768)
Mexico	63 (58–69)	1291 (1184–1413)	452 (423–483)	3675 (3316–4073)	6347 (5658–7051)	34,740 (30,274–39,629)
US	6 (6–6)	12 (12–13)	88 (82–94)	203 (190–217)	2051 (1883–2234)	5141 (4701–5626)
America’s median	42 (6–96)	235 (12–1359)	187 (24–469)	788 (46–3912)	2112 (363–6758)	8790 (679–37,714)
Belgium	42 (39–45)	181 (167–194)	56 (52–61)	252 (229–276)	1595 (1451–1746)	8632 (7763–9591)
France	26 (23–29)	133 (119–151)	30 (27–34)	156 (136–180)	262 (225–304)	1316 (1124–1557)
Germany	7 (6–7)	29 (27–31)	18 (16–21)	66 (57–75)	139 (119–162)	463 (393–541)
Greece	3 (3–3)	13 (12–15)	33 (30–37)	128 (114–143)	636 (552–723)	2219 (1909–2541)
Italy	16 (15–17)	105 (96–115)	44 (38–51)	238 (201–280)	840 (714–983)	4182 (3480–4973)
Portugal	5 (5–6)	44 (40–49)	22 (19–26)	100 (84–120)	141 (118–170)	586 (488–715)
Romania	11 (11–12)	110 (97–124)	21 (19–23)	119 (104–136)	445 (395–499)	1581 (1390–1807)
Slovakia	27 (24–29)	80 (71–89)	32 (28–36)	97 (85–110)	581 (491–677)	1743 (1474–2034)
Spain	4 (4–5)	19 (18–21)	54 (49–59)	513 (476–552)	1063 (911–1238)	41,608 (38,452–45,085)
Sweden	16 (15–17)	40 (38–43)	46 (41–52)	157 (139–175)	332 (286–383)	1152 (996–1321)
Europe Median	13 (3–43)	59 (13–185)	33 (17–58)	140 (63–524)	497 (126–1645)	1647 (436–42,697)
Australia	8 (8–9)	16 (14–17)	46 (41–53)	85 (75–97)	573 (488–676)	1024 (874–1204)
Japan	6 (6–6)	30 (29–32)	50 (47–55)	263 (243–284)	795 (676–935)	4369 (3712–5084)
Malaysia	27 (26–28)	173 (171–192)	72 (67–77)	263 (244–282)	287 (268–307)	1150 (1072–1230)
Singapore	16 (14–17)	205 (183–253)	29 (25–33)	209 (177–247)	464 (391–555)	2974 (2495–3575)
South Korea	2 (2–2)	11 (10–11)	13 (12–13)	65 (60–69)	412 (375–453)	1660 (1512–1820)
Taiwan	13 (12–14)	465 (415–570)	28 (24–32)	482 (407–569)	399 (339–467)	5989 (5037–7088)
Asia-Pacific Median	11 (2–28)	96 (10–510)	37 (12–74)	242 (62–525)	436 (277–862)	2154 (930–6531)
Global Median	13 (2–92)	80 (12–1285)	42 (13–451)	197 (51–3657)	544 (132–6321)	2205 (463–41,490)

Abbreviations: NNV, number needed to vaccinate; PCV13, 13-valent pneumococcal conjugate vaccine; PCV15, 15-valent pneumococcal conjugate vaccine; PCV20, 20-valent pneumococcal conjugate vaccine; US, United States.

## Data Availability

No new data were created or analyzed in this study. Data sharing is not applicable to this article.

## Supplementary Materials

The following supporting information can be downloaded at https://www.mdpi.com/article/10.3390/vaccines14020188/s1, Table S1: Event-specific NNVs by country, PCV20 vs. PCV13 and PCV15 vs PCV13; Table S2: Local Inputs: Detailed local input parameters extracted from published country-specific models.
