# Correction: Dawson et al. A Multi-Country Comparison of Number Needed to Vaccinate for PCV20 and PCV15 in Infants. *Vaccines* 2026, *14*, 188

**DOI:** 10.3390/vaccines14050404

**Published:** 2026-04-30

**Authors:** Euan Dawson, Maria J. Tort, An Ta, Mark H. Rozenbaum

**Affiliations:** 1 Pfizer Inc., Tadworth KT20 7NS, UK; 2 Pfizer Inc., Collegeville, PA 19426, USA; 3 Cytel, Inc., London WC1H 9BB, UK; 4 Pfizer Inc., 2909 LD Capelle a/d Ijssel, The Netherlands

The authors would like to make the following corrections to this published paper [1].

A calculation error was discovered in the calculations of the number needed to vaccinate (NNV) for invasive pneumococcal disease (IPD) in the United States when comparing PCV20 to PCV13. As a result, the NNV estimates for hospitalizations and deaths were revised, leading to updated US-specific results throughout the analysis. The revised tables now reflect these corrected values, and the overall scientific conclusions of the study remain unchanged.

In Table 2 as published, in the US analysis, NNV for hospitalization (PCV20 vs. PCV13) should be 94, not 87; for death, 2488 instead of 2046. In the Americas median, the IQR for hospitalization is (94–192), not (87–192). The median and IQR for deaths is 2269 (not 2053) and 2053–2488 (not 2046–2269). The corrected section in Table 2 appears below.

In Table 3 as published, in the US analysis, the ratio of NNV for hospitalizations should be 2.2 instead of 2.3 and the ratio of NNV for deaths should be 2.1 instead of 2.5. The corrected section in Table 3 appears below.

In Table 4 as published, in the US analysis, the NNV for hospitalization and death in Scenario 1 should be 96.0 and 2498.3 instead of 88.8 and 2051.8, respectively. In the US analysis, the NNV for hospitalization and death in Scenario 2 (a,b) should be (75.2–124.7) and (1989.8–3317.1) instead of (71.1–113.6) and (1702.1–2563.7), respectively. In the US analysis, the NNV for hospitalization and death in Scenario 3 should be 95.2 and 2496.6 instead of 88.4 and 2053.6, respectively. In the US analysis, the NNV for hospitalization and death in Scenario 4 should be 116.4 and 2966.5 instead of 112.5 and 2702.7, respectively. The corrected Table 4 appears below.

In Table 6 as published, in the US analysis, the NNV for hospitalizations was incorrectly reported as 88 (82–94) and should have been 94 (88–101), and the NNV for deaths was incorrectly reported as 2051 (1883–2234) and should have been 2488 (2291–2727). The corrected Table 6 appears below.

In Table S1 as published at https://www.mdpi.com/article/10.3390/vaccines14020188/s1, in the US analysis, the NNV for a case of IPD in PCV20 vs PCV13 should be 854 instead of 503. The corrected Table S1 appears below.

**Table S1:** Event-specific NNVs by country, PCV20 vs. PCV13 and PCV15 vs PCV13.

	Comparison 1: PCV20 vs PCV13				Comparison 2: PCV15 vs PCV13			
Country	IPD	Inpatient Pneumonia	Outpatient Pneumonia	Otitis Media	IPD	Inpatient Pneumonia	Outpatient Pneumonia	Otitis Media
Canada	508	28	59	16	1027	53	102	30
Mexico	13,023	467	198	117	124,907	3777	3554	4514
US	854	106	25	9	1805	229	56	17
Argentina	1077	233	1262	56	6133	1228	8071	317
Chile	6248	198	2655	191	24,569	812	8928	600
Belgium	278	70	184	1397	1583	298	743	4683
France	773	32		176	4387	161		922
Germany	584	19	21	19	2178	67	97	114
Greece	515	35	41	3	2200	136	190	16
Italy	2009	45		25	11,553	242		189
Portugal	735	23		7	5963	100		78
Romania	923	21	43	52	3405	123	3059	2590
Slovakia	2482	32	170	1370	7516	98	509	4160
Spain	623	59	171	4	4612	577	532	21
Sweden	377	53	181	28	1342	176	463	61
Japan	1205	53	10	22	6541	274	51	104
Malaysia	1878	75	173	57	8638	271		531
Singapore	3101	29	57	88	19,763	209		26,711
South Korea	7074	13	3	14	31,260	64	14	143
Taiwan	15,178	28	56	45	280,548	480		14,821
Australia	741	49	242	11	1504	90	439	20

Note: France, Portugal, and Italy publications do not model outpatient NBP. Malaysia, Taiwan and Singapore do not produce NNVs for outpatient NBP in PCV15/13 comparison due to lack of serotype differential in children.

The authors state that the scientific conclusions are unaffected. This correction was approved by the Academic Editor. The original publication has also been updated.

## Figures and Tables

**Table 2:** Number needed to vaccinate to prevent one outcome, for each country and region included for PCV13 vs. PCV20 and PCV13 vs. PCV15.

	Case: PCV13 vs.		Hospitalization: PCV13 vs.		Death: PCV13 vs.	
Country	PCV20	PCV15	PCV20	PCV15	PCV20	PCV15
Argentina	42	235	191	1023	2269	10,581
Canada	8	16	26	51	398	747
Chile	92	328	192	786	2053	8781
Mexico	63	1289	451	3666	6327	34,587
US	6	12	94	203	2488	5128
Americas median (IQR)	42 (8–63)	235 (16–328)	191 (94–192)	786 (203–1023)	2269 (2053–2488)	8781 (5128–10,581)
Belgium	42	180	56	251	1592	8609
France	26	133	30	155	260	1307
Germany	7	29	18	65	138	458
Greece	3	13	33	128	636	2203
Italy	16	105	44	237	834	4162
Portugal	5	44	22	98	139	579
Romania	11	109	21	118	443	1573
Slovakia	26	80	32	97	576	1729
Spain	4	19	53	513	1054	41,593
Sweden	16	40	46	156	332	1144
Europe median (IQR)	13 (5–23)	62 (32–108)	33 (24–46)	142 (103–217)	509 (278–784)	1651 (1185–3672)
Australia	8	15	46	85	568	1018
Japan	6	30	50	263	789	4351
Malaysia	27	173	72	262	286	1149
Singapore	16	205	28	207	459	2941
South Korea	2	11	13	64	411	1654
Taiwan	13	465	28	480	397	5957
Asia-Pacific median (IQR)	11 (7–15)	102 (19–205)	37 (28–49)	235 (115–263)	435 (401–541)	2297 (1275–3998)
Overall median (IQR)	13 (6–26)	80 (19–181)	44 (28–56)	203 (98–263)	568 (397–1054)	2203 (1149–5957)

Abbreviations: IQR, interquartile range; PCV13, 13-valent pneumococcal conjugate vaccine; PCV15, 15-valent pneumococcal conjugate vaccine; PCV20, 20-valent pneumococcal conjugate vaccine; US, United States.

**Table 3:** Ratio of NNV for PCV15 vs. PCV20 by country and outcome, with overall median and IQR.

Country	Case	Hospitalization	Death
Argentina	5.6	5.3	4.7
Canada	1.9	1.9	1.9
Chile	3.5	4.1	4.3
Mexico	20.4	8.1	5.5
US	2.1	2.2	2.1
America’s median	3.5	4.1	4.3
Belgium	4.3	4.5	5.4
France	5.1	5.1	5.0
Germany	4.5	3.6	3.3
Greece	4.7	3.9	3.5
Italy	6.6	5.4	5.0
Portugal	8.6	4.4	4.2
Romania	9.9	5.7	3.6
Slovakia	3.0	3.0	3.0
Spain	4.8	9.6	39.5
Sweden	2.5	3.4	3.4
Europe median	4.7	4.5	3.9
Australia	1.8	1.8	1.8
Japan	5.1	5.2	5.5
Malaysia	6.4	3.7	4.0
Singapore	13.1	7.3	6.4
South Korea	5.2	5.1	4.0
Taiwan	35.3	17.3	15.0
Asia-Pacific median	5.8	5.2	4.8
Overall median (IQR)	5.1 (3.5–6.7)	4.5 (3.6–5.4)	4.2 (3.4–5.4)

Abbreviations: IQR, interquartile range; NNV, number needed to vaccinate; PCV15, 15-valent pneumococcal conjugate vaccine; PCV20, 20-valent pneumococcal conjugate vaccine. Note: NNV ratios are derived from an indirect comparison of PCV20 vs. PCV13 and PCV15 vs. PCV13.

**Table 4:** Scenario analysis of PCV20 NNV to prevent each outcome by country.

PCV20	Scenario 1			Scenario 2 (a,b)			Scenario 3			Scenario 4		
Country	Case	Hospitalization	Death	Case	Hospitalization	Death	Case	Hospitalization	Death	Case	Hospitalization	Death
Australia	8.8	46.7	568.7	6.8–11.3	36.9–61.4	454.5–757.8	8.5	46.4	568.4	10.6	59.6	756.1
Japan	6.1	51.8	789.3	4.7–7.9	40.3–67.1	631.1–1052.1	6.0	50.5	789.1	7.5	62.8	1050.3
US	6.2	96.0	2498.3	4.8–7.9	75.2–124.7	1989.8–3317.1	6.1	95.2	2496.6	7.3	116.4	2966.5
Canada	8.7	26.5	398.6	6.8–11.3	21.2–35.3	318.7–531.4	8.5	26.5	398.6	10.8	35.0	530.9
Italy	15.9	43.9	834.5	12.7–21.1	35.1–57.6	667.3–1109.9	15.9	43.8	834.3	21.0	57.5	1109.7
Germany	6.6	18.3	138.2	5.3–8.4	14.6–24.0	110.8–184.8	6.5	18.2	138.2	8.3	23.7	184.2

Scenario 1: Vaccine effectiveness reduced by 20%, Scenario 2a: Serotype coverage increased by 25%, Scenario 2b: Serotype coverage decreased by 25%, Scenario 3: Duration of protection reduced to 10 years, Scenario 4: Herd effects reduced by 25%.

**Table 6:** Probabilistic central estimates for mean NNV (95% confidence interval) to prevent each outcome by country based on 1000 runs (PCV13 vs. PCV20 and PCV13 vs. PCV15).

	Cases: PCV13 vs.		Hospitalizations: PCV13 vs.		Deaths: PCV13 vs.	
Country	PCV20	PCV15	PCV20	PCV15	PCV20	PCV15
Argentina	42 (39–45)	236 (221–251)	192 (178–206)	1026 (942–1114)	2279 (2074–2513)	10,617 (9482–11,831)
Canada	8 (8–9)	16 (15–17)	27 (13–31)	51 (43–59)	401 (338–470)	752 (631–882)
Chile	93 (87–98)	328 (309–349)	192 (177–208)	789 (727–853)	2060 (1847–2271)	8825 (7895–9768)
Mexico	63 (58–69)	1291 (1184–1413)	452 (423–483)	3675 (3316–4073)	6347 (5658–7051)	34,740 (30,274–39,629)
US	6 (6–6)	12 (12–13)	94 (88–101)	203 (190–217)	2488 (2291–2727)	5141 (4701–5626)
America’s median	42 (6–96)	235 (12–1359)	187 (24–469)	788 (46–3912)	2112 (363–6758)	8790 (679–37,714)
Belgium	42 (39–45)	181 (167–194)	56 (52–61)	252 (229–276)	1595 (1451–1746)	8632 (7763–9591)
France	26 (23–29)	133 (119–151)	30 (27–34)	156 (136–180)	262 (225–304)	1316 (1124–1557)
Germany	7 (6–7)	29 (27–31)	18 (16–21)	66 (57–75)	139 (119–162)	463 (393–541)
Greece	3 (3–3)	13 (12–15)	33 (30–37)	128 (114–143)	636 (552–723)	2219 (1909–2541)
Italy	16 (15–17)	105 (96–115)	44 (38–51)	238 (201–280)	840 (714–983)	4182 (3480–4973)
Portugal	5 (5–6)	44 (40–49)	22 (19–26)	100 (84–120)	141 (118–170)	586 (488–715)
Romania	11 (11–12)	110 (97–124)	21 (19–23)	119 (104–136)	445 (395–499)	1581 (1390–1807)
Slovakia	27 (24–29)	80 (71–89)	32 (28–36)	97 (85–110)	581 (491–677)	1743 (1474–2034)
Spain	4 (4–5)	19 (18–21)	54 (49–59)	513 (476–552)	1063 (911–1238)	41,608 (38,452–45,085)
Sweden	16 (15–17)	40 (38–43)	46 (41–52)	157 (139–175)	332 (286–383)	1152 (996–1321)
Europe Median	13 (3–43)	59 (13–185)	33 (17–58)	140 (63–524)	497 (126–1645)	1647 (436–42,697)
Australia	8 (8–9)	16 (14–17)	46 (41–53)	85 (75–97)	573 (488–676)	1024 (874–1204)
Japan	6 (6–6)	30 (29–32)	50 (47–55)	263 (243–284)	795 (676–935)	4369 (3712–5084)
Malaysia	27 (26–28)	173 (171–192)	72 (67–77)	263 (244–282)	287 (268–307)	1150 (1072–1230)
Singapore	16 (14–17)	205 (183–253)	29 (25–33)	209 (177–247)	464 (391–555)	2974 (2495–3575)
South Korea	2 (2–2)	11 (10–11)	13 (12–13)	65 (60–69)	412 (375–453)	1660 (1512–1820)
Taiwan	13 (12–14)	465 (415–570)	28 (24–32)	482 (407–569)	399 (339–467)	5989 (5037–7088)
Asia-Pacific Median	11 (2–28)	96 (10–510)	37 (12–74)	242 (62–525)	436 (277–862)	2154 (930–6531)
Global Median	13 (2–92)	80 (12–1285)	42 (13–451)	197 (51–3657)	544 (132–6321)	2205 (463–41,490)

Abbreviations: NNV, number needed to vaccinate; PCV13, 13-valent pneumococcal conjugate vaccine; PCV15, 15-valent pneumococcal conjugate vaccine; PCV20, 20-valent pneumococcal conjugate vaccine; US, United States.
