# Supplementary material for: A Multi-Country Comparison of Number Needed to Vaccinate for PCV20 and PCV15 in Infants
Source: Vaccines (Basel). 2026 Feb 18;14(2):188. doi: 10.3390/vaccines14020188 (PMC12945240; doi:10.3390/vaccines14020188)
Supplement: Supplementary file 1 [file vaccines-14-00188-s001.zip › vaccines-4133811-supplementary.pdf]

Supplementary File

Table S1. Event-specific NNVs by country, PCV20 vs. PCV13 and PCV15 vs PCV13

	**Comparison 1: PCV20 vs PCV13**				**Comparison 2: PCV15 vs PCV13**			
**Country**	**IPD**	**Inpatient Pneumonia**	**Outpatient Pneumonia**	**Otitis Media**	**IPD**	**Inpatient Pneumonia**	**Outpatient Pneumonia**	**Otitis Media**
Canada	508	28	59	16	1,027	53	102	30
Mexico	13,023	467	198	117	124,907	3,777	3,554	4,514
US	503	106	25	9	1,805	229	56	17
Argentina	1,077	233	1,262	56	6,133	1,228	8,071	317
Chile	6,248	198	2,655	191	24,569	812	8,928	600
Belgium	278	70	184	1,397	1,583	298	743	4,683
France	773	32		176	4,387	161		922
Germany	584	19	21	19	2,178	67	97	114
Greece	515	35	41	3	2,200	136	190	16
Italy	2,009	45		25	11,553	242		189
Portugal	735	23		7	5,963	100		78
Romania	923	21	43	52	3,405	123	3,059	2,590
Slovakia	2,482	32	170	1370	7,516	98	509	4,160
Spain	623	59	171	4	4,612	577	532	21
Sweden	377	53	181	28	1,342	176	463	61
Japan	1,205	53	10	22	6,541	274	51	104
Malaysia	1,878	75	173	57	8,638	271		531
Singapore	3,101	29	57	88	19,763	209		26,711
South Korea	7,074	13	3	14	31,260	64	14	143
Taiwan	15,178	28	56	45	280,548	480		14,821
Australia	741	49	242	11	1504	90	439	20

Note: France, Portugal, and Italy publications do not model outpatient NBP. Malaysia, Taiwan and Singapore do not produce NNVs for outpatient NBP in PCV15/13 comparison due to lack of serotype differential in children.

Table S2. Local inputs

The purpose of this document is to provide a comprehensive and transparent inventory of data for parameters included in the modelling of the 21 countries included in this manuscript. The document will present the data for the following inputs: **(1)** Outline of the direct effect inputs which are common among countries with slight differences depending on schedule, **(2)** Outline of the indirect effect inputs which are common among certain groups of countries with slight differences between groups. **(3)** Local data for each individual country, relating to the epidemiological, population, and vaccine uptake parameters.

(1) Direct Vaccine Effectiveness Inputs

<table>
<tr><th colspan="2">Direct effects, %</th><th>IPD</th><th>Hospitalized pneumonia</th><th>Non-hospitalized pneumonia</th><th>Otitis media</th></tr>
<tr><td colspan="2">All vaccines under 3+1 schedule [a]</td><td>86.0</td><td rowspan="4">25.5</td><td rowspan="4">6.0</td><td rowspan="4">7.8</td></tr>
<tr><td colspan="2">All vaccines under 2+1 schedule [b]</td><td>88.7</td></tr>
<tr><td rowspan="2">Mixed schedules [c]:
PCV13/PCV15 2+1
PCV20 3+1</td><td>2+1 schedule</td><td>78.2</td></tr>
<tr><td>3+1 schedule</td><td>89.7</td></tr>
</table>

[a]Countries included: The US, Japan, Canada, and South Korea
[b]Countries included: Mexico, Argentina, Portugal, Sweden, Australia, Malaysia, Singapore, Italy, Greece
[c]Countries included: Belgium, France, Germany, Romania, Spain, Taiwan, Chile, Slovakia

Year	Vaccine Waning
**Year 1-5**	0%
**Year 6**	10%
**Year 7**	19%
**Year 8**	27%
**Year 9**	34%
**Year 10**	41%
**Year 11**	47%

**Year 12**	52%
**Year 13**	57%
**Year 14**	61%
**Year 15**	65%
**Year 16-25**	100%

(2) Indirect Vaccine Effectiveness Inputs

Countries: Australia, Belgium, Germany, Greece, Malaysia, Portugal, Romania, Singapore, Slovakia, Spain	IPD	Hospitalized pneumonia	Non-hospitalized pneumonia	Otitis media
**Indirect effect – maximum reduction, %**				
<5 y	83.0	43.8	32.3	28.0
5–17 y	83.0	35.6	26.2	28.0
18–34 y	88.0	22.5	0.0	-
35–49 y	88.0	22.5	0.0	-
50-64 y	77.0	25.2	0.0	-
≥65 y	73.0	26.9	0.0	-

	Year(s)					
	**1**	**2**	**3**	**4**	**5**	**6–10**
**Indirect effect – ramp-up (PCV15/PCV20), %**	0.0	37.5	52.8	67.7	82.7	100.0

Country: France	IPD	Hospitalized pneumonia	Non-hospitalized pneumonia	Otitis media
**Indirect effect – maximum reduction, %**				
<5 y	99.4	43.8	32.3	28.0
5–17 y	99.4	35.6	26.2	28.0
18–34 y	96.9	22.5	0.0	-
35–49 y	96.9	22.5	0.0	-
50-64 y	96.9	25.2	0.0	-
≥65 y	80.8	26.9	0.0	-

	**Year(s)**					
	**1**	**2**	**3**	**4**	**5**	**6–10**
**Indirect effect – ramp-up (PCV15/PCV20), %**	0.0	37.5	52.8	67.7	82.7	100.0

Country: Italy	IPD	Hospitalized pneumonia	Non-hospitalized pneumonia	Otitis media
**Indirect effect – maximum reduction, %**				
<5 y	83.0	30.5	N/A	20.0
5–17 y	83.0	30.5	N/A	20.0
18–34 y	88.0	15.0	N/A	-
35–49 y	88.0	15.0	N/A	-
50-64 y	77.0	15.0	N/A	-
≥65 y	73.0	15.0	N/A	-

	**Year(s)**					
	**1**	**2**	**3**	**4**	**5**	**6–10**
**Indirect effect – ramp-up (PCV15/PCV20), %**	0.0	37.5	52.8	67.7	82.7	100.0

Countries: Japan, South Korea	IPD	Hospitalized pneumonia	Non-hospitalized pneumonia	Otitis media
**Indirect effect – maximum reduction, %**				
<5 y	93.1	29.4	29.4	12.0
5–17 y	93.1	29.4	29.4	12.0
18–34 y	89.2	30.3	30.3	-
35–49 y	90.1	37.8	37.8	-
50-64 y	90.1	24.8	24.8	-
≥65 y	88.2	6.3	6.3	-

	Year(s)					
	**1**	**2**	**3**	**4**	**5**	**6–10**
**Indirect effect – ramp-up (PCV15/PCV20), %**	0.0	37.5	52.8	67.7	82.7	100.0

Country: Sweden	IPD	Hospitalized pneumonia	Non-hospitalized pneumonia	Otitis media
**Indirect effect – maximum reduction, %**				
<5 y	83	43.8	32.3	24.9
5–17 y	83	35.6	26.2	24.9
18–34 y	88	22.5	0.0	-
35–49 y	88	22.5	0.0	-
50-64 y	77	25.2	0.0	-
≥65 y	73	26.9	0.0	-

	Year(s)					
	**1**	**2**	**3**	**4**	**5**	**6–10**
**Indirect effect – ramp-up (PCV15/PCV20), %**	0.0	37.5	52.8	67.7	82.7	100.0

Country: Taiwan	IPD	Hospitalized pneumonia	Non-hospitalized pneumonia	Otitis media
**Indirect effect – maximum reduction, %**				
<5 y	86.7 – 93.5	43.8	32.3	28.0
5–17 y	85	35.6	26.2	28.0
18–34 y	57.9	22.5	0.0	-
35–49 y	81.6	22.5	0.0	-
50-64 y	52.7	25.2	0.0	-
≥65 y	73	26.9	0.0	-

	Year(s)					
	**1**	**2**	**3**	**4**	**5**	**6–10**
**Indirect effect – ramp-up (PCV15/PCV20), %**	0.0	37.5	52.8	67.7	82.7	100.0

USA	IPD	Hospitalized pneumonia	Non-hospitalized pneumonia	Otitis media
**Indirect effect – maximum reduction, %**				
<5 y	93.1	30.3	30.3	12.5
5–17 y	93.1	30.3	30.3	12.5
18–34 y	89.2	37.8	37.8	-
35–49 y	90.1	37.8	37.8	-
50-64 y	90.1	24.8	24.8	-
≥65 y	88.2	6.3	6.3	-

	Year(s)					
	**1**	**2**	**3**	**4**	**5**	**6–10**
**Indirect effect – ramp-up (PCV15/PCV20), %**	0.0	37.5	52.8	67.7	82.7	100.0

<table>
<tr><th>Countries: Argentina, Chile</th><th colspan="2">IPD</th><th colspan="2">Hospitalized pneumonia</th><th colspan="2">Non-hospitalized pneumonia</th><th>Otitis media</th></tr>
<tr><td colspan="8">Indirect effect – maximum reduction, %</td></tr>
<tr><td><5 y</td><td colspan="2">83</td><td colspan="2">43.8</td><td colspan="2">32.3</td><td>28.0</td></tr>
<tr><td>5–17 y</td><td colspan="2">83</td><td colspan="2">35.6</td><td colspan="2">26.2</td><td>28.0</td></tr>
<tr><td>18–34 y</td><td colspan="2">27</td><td colspan="2">22.5</td><td colspan="2">0.0</td><td>-</td></tr>
<tr><td>35–49 y</td><td colspan="2">27</td><td colspan="2">22.5</td><td colspan="2">0.0</td><td>-</td></tr>
<tr><td>50-64 y</td><td colspan="2">27</td><td colspan="2">25.2</td><td colspan="2">0.0</td><td>-</td></tr>
<tr><td>≥65 y</td><td colspan="2">27.2</td><td colspan="2">26.9</td><td colspan="2">0.0</td><td>-</td></tr>
<tr><td></td><td colspan="7">Year(s)</td></tr>
<tr><td></td><td>1</td><td colspan="2">2</td><td>3</td><td>4</td><td>5</td><td>6–10</td></tr>
<tr><td>Indirect effect – ramp-up (PCV15/PCV20), %</td><td>0.0</td><td colspan="2">37.5</td><td>52.8</td><td>67.7</td><td>82.7</td><td>100.0</td></tr>
</table>

<table>
<tr><th>Country: Canada</th><th colspan="2">IPD</th><th colspan="2">Hospitalized pneumonia</th><th colspan="2">Non-hospitalized pneumonia</th><th>Otitis media</th></tr>
<tr><td colspan="8">Indirect effect – maximum reduction, %</td></tr>
<tr><td><5 y</td><td colspan="2">81</td><td colspan="2">43.8</td><td colspan="2">32.3</td><td>28.0</td></tr>
<tr><td>5–17 y</td><td colspan="2">81</td><td colspan="2">35.6</td><td colspan="2">26.2</td><td>28.0</td></tr>
<tr><td>18–34 y</td><td colspan="2">65</td><td colspan="2">22.5</td><td colspan="2">0.0</td><td>-</td></tr>
<tr><td>35–49 y</td><td colspan="2">65</td><td colspan="2">22.5</td><td colspan="2">0.0</td><td>-</td></tr>
<tr><td>50-64 y</td><td colspan="2">74</td><td colspan="2">25.2</td><td colspan="2">0.0</td><td>-</td></tr>
<tr><td>≥65 y</td><td colspan="2">81</td><td colspan="2">26.9</td><td colspan="2">0.0</td><td>-</td></tr>
<tr><td></td><td colspan="7">Year(s)</td></tr>
<tr><td></td><td>1</td><td colspan="2">2</td><td>3</td><td>4</td><td>5</td><td>6–10</td></tr>
<tr><td>Indirect effect – ramp-up (PCV15/PCV20), %</td><td>0.0</td><td colspan="2">37.5</td><td>52.8</td><td>67.7</td><td>82.7</td><td>100.0</td></tr>
</table>

Country: Mexico	IPD		Hospitalized pneumonia		Non-hospitalized pneumonia	Otitis media
**Indirect effect – maximum reduction, %**						
<5 y	50.3		43.8		32.3	28.0
5–17 y	50.3		35.6		26.2	28.0
18–34 y	57.8		22.5		0.0	-
35–49 y	48.9		22.5		0.0	-
50-64 y	51.2		25.2		0.0	-
≥65 y	69.1		26.9		0.0	-
	**Year(s)**					
	**1**	**2**	**3**	**4**	**5**	**6–10**
**Indirect effect – ramp-up (PCV15/PCV20), %**	0.0	37.5	52.8	67.7	82.7	100.0

(3) Individual Country Local Data

Argentina [1]	Age group							
	<12 mo	12–23 mo	24–59 mo	5–17 yrs	18–34 yrs	35–49 yrs	50–64 yrs	≥65 yrs
**Population size**								
No. of persons	712,512	715,342	2,166,123	9,459,948	11,544,733	9,433,454	6,685,891	5,898,228
**Serotype coverage, %**								
PCV13	30.5	30.5	30.5	48.4	38.8	35.2	41.1	39.7
PCV15	34.2	34.2	34.2	51.5	41.6	36.5	45.7	45.7
PCV20	51.6	51.6	51.6	69.7	67.2	59.8	68.9	62.4
**Disease incidence per 100,000 individuals**								
IPD	10.0	10.0	10.0	3.3	3.3	27.7	60.6	143.3
Hospitalized pneumonia	772.5	285.8	189.6	16.1	16.1	88.0	181.9	709.9
Non-hospitalized pneumonia	288.6	106.8	70.8	100.7	100.7	157.3	245.8	969
Otitis media	8,943	7,690.5	7,690.5	-	-	-	-	-
**Proportion of IPD cases, %**								

Meningitis	38.1	21.4	13.0	23.90	25.0	20.0	38.9	18.0
**Fatality rate, %**								
Meningitis	14.3	14.3	14.3	14.3	15.0	15.0	15.0	15.0
Bacteremia	1.50	1.50	1.50	1.5	3.20	9.0	15.20	26.6
Hospitalized pneumonia	3.50	3.5	1.5	1.0	0.4	3.8	8.4	14.1
**Other Inputs**								
**Vaccine uptake, %**								
PCV13/15/20	78.92							
**Incoming birth cohort**								
No. of persons	636,838 - 686,860							

**Australia [2]**	**Age group**							
	**<12 mo**	**12–23 mo**	**24–59 mo**	**5–17 yrs**	**18–34 yrs**	**35–49 yrs**	**50–64 yrs**	**≥65 yrs**
**Population size**								
No. of persons	305,057	309,895	916,853	4,265,548	6,117,122	5,323,741	4,755,729	4,588,235
**Serotype coverage, %**								
PCV13	23.7	23.7	54.0	56.9	37.0	37.1	35.3	27.2
PCV15	44.9	44.9	61.9	67.7	47.2	48.9	48.9	44.1
PCV20	59.3	59.3	70.8	80.0	82.4	68.6	63.9	56.1
**Disease incidence per 100,000 individuals**								
IPD	33.4	33.4	19.2	4.2	2.6	5.6	9.8	17.1
Hospitalized pneumonia	422.9	807.3	807.3	66.0	64.7	130.5	311.1	1,473.8
Non-hospitalized pneumonia	1950.6	1950.6	903.2	94.3	108.2	87.8	179.2	839
Otitis media	44,931.2	44,202.6	25,514.3	1,646.2	-	-	-	-
**Proportion of IPD cases, %**								
Meningitis	8.96	8.96	8.14	21.76	0.71	0	0.66	1.42
Bacteremia	91.04	91.04	91.86	78.22	99.29	100	99.34	98.58
**Fatality rate, %**								
Meningitis	2	2	0.3	0.3	7.4	7.4	7.4	11.4
Bacteremia	1	1	0.3	0.3	7.4	7.4	7.4	11.4
Hospitalized pneumonia	1	1	0.3	0.3	7.4	7.4	7.4	11.4

**Other Inputs**	
**Vaccine uptake, %**	
PCV13/15/20	95.0
**Incoming birth cohort**	
No. of persons	314,750 – 358,050

**Belgium [3]**	**Age group**							
	**<12 mo**	**12–23 mo**	**24–59 mo**	**5–17 yrs**	**18–34 yrs**	**35–49 yrs**	**50–64 yrs**	**≥65 yrs**
**Population size**								
No. of persons	117,375	117,375	352,125	1,740,051	2,465,420	2,279,003	2,335,638	2,356,665
**Serotype coverage, %**								
PCV13	17.2	17.2	20.2	20.2	43.7	43.7	29.4	28
PCV15	33.6	33.6	30.3	30.3	47	47	36	36.5
PCV20	57.5	57.5	63.5	63.5	79.5	79.5	71.9	64.1
**Disease incidence per 100,000 individuals**								
IPD	58.4	58.4	11.2	11.2	15.7	15.7	15.7	35.2
Hospitalized pneumonia	1,161	228	228	113.6	58.1	92.4	145	869.2
Non-hospitalized pneumonia	288.7	288.7	288.7	511.2	247.3	309.8	390.9	941
Otitis media	339	57.5	57.5	32.6				
**Proportion of IPD cases, %**								
Meningitis	13.8	3.5	3.5	9.15	6.29	6.29	6.29	4.65
Bacteremia								
**Fatality rate, %**								
Meningitis	6.6	11.3	11.3	2.66	8.68	8.91	7.73	23.22
Bacteremia	1	0	0	1.5	9.69	15.8	13.43	23.19
Hospitalized pneumonia	0.04	0	0	0.49	0.25	0.66	1.04	2.43
**Other Inputs**								
**Vaccine uptake, %**								
PCV13/15/20	95.4							
**Incoming birth cohort**								
No. of persons	117,071 – 119,805							

Canada [4]	Age group							
	<12 mo	12–23 mo	24–59 mo	5–17 yrs	18–34 yrs	35–49 yrs	50–64 yrs	≥65 yrs
**Population size**								
No. of persons	358,001	369,839	1,154,731	5,347,599	8,635,962	7,560,784	7,737,400	7,081,792
**Serotype coverage, %**								
PCV13	16.6	16.6	27.5	28.4	41.2	41.2	30.3	26.5
PCV15	37.8	37.8	43.8	48.9	52.0	52.0	42.7	41.7
PCV20	50.7	50.7	61.2	61.4	72.5	72.5	60.9	54.4
**Disease incidence per 100,000 individuals**								
IPD	13.2	13.2	11.2	2.0	4.3	4.3	10.3	21.2
Hospitalized pneumonia	357	357	213	61	96	96	342	1860
Non-hospitalized pneumonia	2973	2973	2725	994	849	849	1594	5004
Otitis media	17,107	17,107	18,955	-	-	-	-	-
**Proportion of IPD cases, %**								
Meningitis	7	7	7	7	7	7	7	7
Bacteremia	93	93	93	93	93	93	93	93
**Fatality rate, %**								
Meningitis	5.99	5.99	1.85	6.41	5.07	5.07	10.73	13.88
Bacteremia	5.99	5.99	1.85	6.41	5.07	5.07	10.73	13.88
Hospitalized pneumonia	0.44	0.44	0.2	0.3	1.4	1.4	3.8	7.97
**Other Inputs**								
**Vaccine uptake, %**								
PCV13/15/20	84.0							
**Incoming birth cohort**								
No. of persons	353,000 – 360,900							

Chile [5]	Age group							
	<12 mo	12–23 mo	24–59 mo	5–17 yrs	18–34 yrs	35–49 yrs	50–64 yrs	≥65 yrs
**Population size**								
No. of persons	235,715	236,930	711,436	3,278,003	5,003,220	4,320,039	3,531,847	2,769,187
**Serotype coverage, %**								

PCV13	40.7	42	45.2	46.9	44.4	42.9	42.4	42.7
PCV15	47.2	50.3	49.3	47.9	46.2	46.4	45	47.8
PCV20	63.5	62.2	59.4	57.9	60.1	61.5	61.9	61.7
**Disease incidence per 100,000 individuals**								
IPD	13	15.7	8.4	1.5	1.0	2.3	5.2	10.0
Hospitalized pneumonia	575.8	696.9	372.1	65	42.8	100.4	228.3	483.2
Non-hospitalized pneumonia	215.1	260.4	139	24.3	16	37.5	85.3	181
Otitis media	3,236	3,236	3,236	-	-	-	-	-
**Proportion of IPD cases, %**								
Meningitis	22.6	3	5.5	21.1	30.4	20.2	17.7	13.6
Bacteremia								
**Fatality rate, %**								
Meningitis	15.2	15	16.7	9.9	9.9	9.9	9.9	9.9
Bacteremia	39.5	18	20	40	40	40	40	40
Hospitalized pneumonia	9.1	3.1	3.4	2.9	0	0	15	15
**Other Inputs**								
**Vaccine uptake, %**								
PCV13/15/20	98.0							
**Incoming birth cohort**								
No. of persons	237,533 – 246,711							

**France [6]**	**Age group**							
	**<12 mo**	**12–23 mo**	**24–59 mo**	**5–17 yrs**	**18–34 yrs**	**35–49 yrs**	**50–64 yrs**	**≥65 yrs**
**Population size**								
No. of persons	713,000	685,044	2,138,078	10,802,386	13,439,124	12,720,252	13,227,533	14,725,470
**Serotype coverage, %**								
PCV13	14.00	14.00	19.00	17.00	28.00	28.00	28.00	34.00
PCV15	24.00	24.00	24.00	23.0	32.00	32.00	32.00	40.00
PCV20	50.00	50.00	54.00	54.00	63.00	63.00	63.00	62.50
**Disease incidence per 100,000 individuals**								
IPD	14.50	4.60	4.60	1.70	3.90	3.90	3.90	15.20

Hospitalized pneumonia	967.03	634.1	337.44	76.98	76.98	117.90	395.30	1,916.70
Non-hospitalized pneumonia	-	-	-	-	-	-	-	-
Otitis media	847.00	2,099.00	1,731.00	-	-	-	-	-
**Proportion of IPD cases, %**								
Meningitis	40.52	40.52	19.45	35.80	35.80	41.76	41.76	23.50
Bacteremia								
**Fatality rate, %**								
Meningitis	6.00	6.00	6.00	6.00	12.2	12.20	16.60	27.30
Bacteremia	1.50	1.50	1.50	1.50	8.40	8.40	15.70	21.40
Hospitalized pneumonia	0.80	0.80	0.80	0.80	4.70	4.70	11.20	15.20
**Other Inputs**								
**Vaccine uptake, %**								
PCV13/15/20	95.70							
**Incoming birth cohort**								
No. of persons	707,000 – 714,000							

**Germany [7]**	**Age group**							
	**<12 mo**	**12–23 mo**	**24–59 mo**	**5–17 yrs**	**18–34 yrs**	**35–49 yrs**	**50–64 yrs**	**≥65 yrs**
**Population size**								
No. of persons	791,254	780,795	2,403,284	9,887,926	16,539,516	15,331,897	19,065,953	18,972,098
**Serotype coverage, %**								
PCV13	12.9	12.9	20.5	29.7	27.8	27.8	30.3	30.9
PCV15	17.8	17.8	28.2	40.5	34.2	34.2	38.9	40.4
PCV20	47.5	47.5	61.5	70.3	68.7	68.7	67	61.1
**Disease incidence per 100,000 individuals**								
IPD	15.8	15.8	1.4	1	1.7	1.7	10.6	22
Hospitalized pneumonia	1,493	814	814	132.2	111.4	111.4	538.1	2,549.5
Non-hospitalized pneumonia	2,447	2,447	8,492	2,426	401.3	401.3	691.3	1,022
Otitis media	14,749	14,749	17,939	-	-	-	-	-
**Proportion of IPD cases, %**								
Meningitis	33.4	33.4	33.4	33.4	9.1	9.1	4.6	1.8

Bacteremia								
**Fatality rate, %**								
Meningitis	6.8	10.70	6.9	4.81	8.74	8.74	12.95	19.65
Bacteremia	1	2.2	2.3	7.23	8.74	8.74	12.95	19.65
Hospitalized pneumonia	0.15	0.09	0.09	0.9	4.42	4.42	11.94	18.67
**Other Inputs**								
**Vaccine uptake, %**								
PCV13/15/20	89.8							
**Incoming birth cohort**								
No. of persons	723,600 – 752,300							

**Greece [8]**	**Age group**							
	**<12 mo**	**12–23 mo**	**24–59 mo**	**5–17 yrs**	**18–34 yrs**	**35–49 yrs**	**50–64 yrs**	**≥65 yrs**
**Population size**								
No. of persons	107,137	107,137	322,968	1,352,882	2,450,762	2,393,924	2,017,623	2,108,162
**Serotype coverage, %**								
PCV13	12.9	12.9	20.5	29.7	27.8	27.8	30.3	30.9
PCV15	17.8	17.8	28.2	40.5	34.2	34.2	38.9	40.4
PCV20	47.5	47.5	61.5	70.3	68.7	68.7	67	61.1
**Disease incidence per 100,000 individuals**								
IPD	12	12	5	2	3	6	12	22
Hospitalized pneumonia	585	585	293	94	123	123	476	1500
Non-hospitalized pneumonia	2900	2900	3300	1200	620	620	1594	2800
Otitis media	63500	63500	38000	11765	-	-	-	-
**Proportion of IPD cases, %**								
Meningitis	10.2	10.2	10.2	10.2	6.17	8.5	8.67	9.33
Bacteremia								
**Fatality rate, %**								
Meningitis	5.99	5.99	1.85	6.41	5.07	5.07	10.73	13.88
Bacteremia	5.99	5.99	1.85	6.41	5.07	5.07	10.73	13.88
Hospitalized pneumonia	0.44	0.44	0.2	0.3	1.4	1.4	3.8	7.97

**Other Inputs**	
**Vaccine uptake, %**	
PCV13/15/20	84
**Incoming birth cohort**	
No. of persons	76,541

**Italy [9]**	**Age group**							
	**<12 mo**	**12–23 mo**	**24–59 mo**	**5–17 yrs**	**18–34 yrs**	**35–49 yrs**	**50–64 yrs**	**≥65 yrs**
**Population size**								
No. of persons	395,348	402,454	1,283,666	6,983,122	10,293,593	11,593,576	13,721,513	14,155,573
**Serotype coverage, %**								
PCV13	10.7	10.7	10.7	26.9	26.9	26.9	26.9	32.6
PCV15	16.5	16.5	16.5	31.1	31.1	31.1	31.1	40.3
PCV20	53.5	53.5	53.5	65.8	65.8	65.8	65.8	70.2
**Disease incidence per 100,000 individuals**								
IPD	6.2	1.3	1.3	0.6	0.2	1	1	4.4
Hospitalized pneumonia	189	310.5	310.5	81	78	140	140	1311
Non-hospitalized pneumonia	-	-	-	-	-	-	-	-
Otitis media	18200	18200	18200	-	-	-	-	-
**Proportion of IPD cases, %**								
Meningitis	48	32	32	67	44	30	30	14
Bacteremia								
**Fatality rate, %**								
Meningitis	4	4	4	3.5	11	15	15	32
Bacteremia	4	1	1	0	16	20	26	34
Hospitalized pneumonia	1.3	0.5	0.4	1.3	0.26	0.21	0.83	6.32
**Other Inputs**								
**Vaccine uptake, %**								
PCV13/15/20	90.58							
**Incoming birth cohort**								
No. of persons	383,414 – 394,004							

Japan [10]	Age group							
	<12 mo	12–23 mo	24–59 mo	5–17 yrs	18–34 yrs	35–49 yrs	50–64 yrs	≥65 yrs
**Population size**								
No. of persons	830,000	836,000	2,724,000	13,665,000	21,508,000	25,256,000	24,468,000	36,128,000
**Serotype coverage, %**								
PCV13	3.1	3.1	3.1	3.1	29.2	29.2	29.2	29.2
PCV15	10.3	10.3	10.3	10.3	35	35	35	35
PCV20	37.7	37.7	37.7	37.7	61.1	61.1	61.1	61.1
**Disease incidence per 100,000 individuals**								
IPD	11	10.2	10.2	0.5	0.3	0.9	2	5.5
Hospitalized pneumonia	2,353.7	2353.7	909.6	141.1	75.1	75.1	219	1225.6
Non-hospitalized pneumonia	7808.2	7808.2	8694.4	2231.1	1009	1009	1659.2	2396
Otitis media	28,903.8	28,903.8	28,903.8	-	-	-	-	-
**Proportion of IPD cases, %**								
Meningitis	6	6	6	6	6	6	6	6
Bacteremia	94	94	94	94	94	94	94	94
**Fatality rate, %**								
Meningitis	3.7	3.7	3.7	3.7	7.1	7.1	7.1	12.1
Bacteremia	4	4	4	4	13.8	13.8	13.8	19.2
Hospitalized pneumonia	0.08	0.08	0.08	0.17	0.26	1.33	2.58	16.87
**Other Inputs**								
**Vaccine uptake, %**								
PCV13/15/20	97.9							
**Incoming birth cohort**								
No. of persons	772,651 – 798,000							

Malaysia [11]	Age group							
	<12 mo	12–23 mo	24–59 mo	5–17 yrs	18–34 yrs	35–49 yrs	50–64 yrs	≥65 yrs
**Population size**								
No. of persons	473,300	473,300	1,419,000	6,867,280	9,966,120	7,277,900	4,438,800	2,485,200
**Serotype coverage, %**								

PCV13	87.6	87.6	87.6	87.6	77.3	77.3	77.3	77.3
PCV15	87.6	87.6	87.6	87.6	78.1	78.1	78.1	78.1
PCV20	91.8	91.8	91.8	91.8	82.1	82.1	82.1	82.1
**Disease incidence per 100,000 individuals**								
IPD	45	45	45	7.2	7.6	8	16.9	48.2
Hospitalized pneumonia	791.8	791.8	759	882.9	1009.9	1009.9	4383.4	6676.8
Non-hospitalized pneumonia	-	-	-	-	-	-	-	-
Otitis media	19786	19786	22149	5696	-	-	-	-
**Proportion of IPD cases, %**								
Meningitis	35	35	12	10	8.99	8.92	6.98	1.99
Bacteremia								
**Fatality rate, %**								
Meningitis	33	33	33	10	10	11	11.4	23.8
Bacteremia	23	23	23	23	13.1	13.1	13.1	13.1
Hospitalized pneumonia	26.48	26.48	30.58	39.78	23.85	23.85	28.05	27.85
**Other Inputs**								
**Vaccine uptake, %**								
PCV13/15/20	95							
**Incoming birth cohort**								
No. of persons	463,200 – 517,646							

**Mexico [12]**	**Age group**							
	**<12 mo**	**12–23 mo**	**24–59 mo**	**5–17 yrs**	**18–34 yrs**	**35–49 yrs**	**50–64 yrs**	**≥65 yrs**
**Population size**								
No. of persons	2,194,390	2,191,694	6,579,708	28,551,145	35,300,147	25,801,680	18,458,888	10,732,982
**Serotype coverage, %**								
PCV13	29.0	45.4	33.6	28.1	29.1	38.3	36.1	23.0
PCV15	30.8	45.4	33.6	28.5	31.6	39.2	39.2	26.9
PCV20	37.5	51.9	49.8	44.7	46.3	54.1	50.5	39.7
**Disease incidence per 100,000 individuals**								
IPD	18.6	7.3	4.1	2.0	0.6	0.9	2.1	2.5

Hospitalized pneumonia	801.5	271.1	271.1	42.9	26.8	54.1	128.1	370.5
Non-hospitalized pneumonia	6412.0	2168.9	2168.9	343.4	214.1	433.0	638.7	654.9
Otitis media	855.3	855.3	1081.8	1860.6	-	-	-	-
**Proportion of IPD cases, %**								
Meningitis	30.43	30.43	30.43	14.29	0.00	0.00	38.90	38.90
Bacteremia	69.57	69.57	69.57	85.71	100.00	100.00	61.10	61.10
**Fatality rate, %**								
Meningitis	14.7	14.7	14.7	14.7	14.7	14.7	20.0	25.3
Bacteremia	4.5	4.5	3.5	4.2	4.1	4.1	4.1	4.1
Hospitalized pneumonia	3.0	3.0	3.0	3.0	3.0	3.0	12.4	16.8
**Other Inputs**								
**Vaccine uptake, %**								
PCV13/15/20	83.5							
**Incoming birth cohort**								
No. of persons	2,142,552 – 2,189,959							

**Portugal [13]**	**Age group**							
	**<12 mo**	**12–23 mo**	**24–59 mo**	**5–17 yrs**	**18–34 yrs**	**35–49 yrs**	**50–64 yrs**	**≥65 yrs**
**Population size**								
No. of persons	85,570	85,570	256,710	1,247,866	1,892,702	2,181,748	2,237,234	2,493,506
**Serotype coverage, %**								
PCV13	28.3	6.5	53.5	55.8	37	37	35.7	36.8
PCV15	36.7	19.6	56.3	55.8	39.7	39.7	39.5	45.9
PCV20	70	63	77.5	74.4	75.5	75.5	75.3	71.9
**Disease incidence per 100,000 individuals**								
IPD	20.5	23.4	11.5	19.5	2.8	2.1	3	5.6
Hospitalized pneumonia	556	556	293	55	50.8	63.7	250	1712.8
Non-hospitalized pneumonia	-	-	-	-	-	-	-	-
Otitis media	24,290	24,290	24,290	17,294	-	-	-	-
**Proportion of IPD cases, %**								
Meningitis	17	11.36	11.36	11.36	22.62	22.62	25.55	17.46

Bacteremia								
**Fatality rate, %**								
Meningitis	1.7	1.7	1.7	1.7	6	6	6	6
Bacteremia	1.7	1.7	1.7	1.7	4.26	4.26	3.8	5.14
Hospitalized pneumonia	1.7	1.7	1.7	1.7	5.37	5.37	11.04	20.60
**Other Inputs**								
**Vaccine uptake, %**								
PCV13/15/20	98							
**Incoming birth cohort**								
No. of persons	83,664							

**Romania [14]**	**Age group**							
	**<12 mo**	**12–23 mo**	**24–59 mo**	**5–17 yrs**	**18–34 yrs**	**35–49 yrs**	**50–64 yrs**	**≥65 yrs**
**Population size**								
No. of persons	184,950	178,900	594,750	2,781,710	3,493,440	4,173,640	3,882,490	3,760,130
**Serotype coverage, %**								
PCV13	63.9	63.9	64.4	66.9	54.9	54.9	54.9	54.9
PCV15	63.9	63.9	64.4	67.5	59.2	59.2	59.2	59.2
PCV20	75	75	80	79.8	69	69	69	69
**Disease incidence per 100,000 individuals**								
IPD	19	19	5	1	5	5	5	22
Hospitalized pneumonia	4,570	4,570	4,570	732	276	420	1,082	2,560
Non-hospitalized pneumonia	12,263	12,263	7,307	2,261	2,496	1,897	2,013	2,266
Otitis media	7981.1	7981.1	7813.4	1766	-	-	-	-
**Proportion of IPD cases, %**								
Meningitis	26.20	26.20	11.13	53.51	22.82	22.82	22.82	5.29
Bacteremia	73.80	73.80	88.87	46.49	77.18	77.18	77.18	94.71
**Fatality rate, %**								
Meningitis	0.8	0.8	0.8	0.8	11.4	11.4	17.5	25.8
Bacteremia	1.5	1.5	1.5	1.5	7.9	7.9	13.0	20.0
Hospitalized pneumonia	1.6	1.6	1.6	0.4	4.5	4.5	10.0	7.9

Other Inputs	
**Vaccine uptake, %**	
PCV13/15/20	85.0
**Incoming birth cohort**	
No. of persons	175,735 – 196,652

**Singapore [15]**	**Age group**							
	**<12 mo**	**12–23 mo**	**24–59 mo**	**5–17 yrs**	**18–34 yrs**	**35–49 yrs**	**50–64 yrs**	**≥65 yrs**
**Population size**								
No. of persons	30.052	34,223	110,993	533,509	913,085	912,725	896,823	717,843
**Serotype coverage, %**								
PCV13	59.5	59.5	59.5	59.5	58.1	58.1	58.1	58.1
PCV15	59.6	59.6	59.6	59.6	60	60	60	60
PCV20	65.6	65.6	65.6	65.6	70.1	70.1	70.1	70.1
**Disease incidence per 100,000 individuals**								
IPD	0.4	0.4	0.6	0.4	0.5	1.4	3.4	8.8
Hospitalized pneumonia	2619.2	1528.6	1645	1057.5	162	238.7	596.3	4116.8
Non-hospitalized pneumonia	9706	8603.8	10646.4	8332.5	1403.7	1442	2286.2	9100
Otitis media	9866.3	11749.3	11564.2	2653.7	-	-	-	-
**Proportion of IPD cases, %**								
Meningitis	7	7	7	7	7	7	7	7
Bacteremia	93	93	93	93	93	93	93	93
**Fatality rate, %**								
Meningitis	7	5.3	3.4	4.6	4.9	7.9	11.1	14.2
Bacteremia	7	5.3	3.4	4.6	4.9	7.9	11.1	14.2
Hospitalized pneumonia	1.3	0.5	0.4	1.3	1.4	1.4	3.8	8
**Other Inputs**								
**Vaccine uptake, %**								
PCV13/15/20	90.19							
**Incoming birth cohort**								
No. of persons	33,541							

Slovakia [16]	Age group							
	**<12 mo**	**12–23 mo**	**24–59 mo**	**5–17 yrs**	**18–34 yrs**	**35–49 yrs**	**50–64 yrs**	**≥65 yrs**
**Population size**								
No. of persons	51,198	55,672	176,100	749,430	1,058,383	1,301,082	1,052,626	982,250
**Serotype coverage, %**								
PCV13	53.4	53.4	53.4	53.4	53.4	53.4	53.4	53.4
PCV15	61.2	61.2	61.2	61.2	61.2	61.2	61.2	61.2
PCV20	76.7	76.7	76.7	76.7	76.7	76.7	76.7	76.7
**Disease incidence per 100,000 individuals**								
IPD	5.3	2.5	2.5	0.4	0.4	0.4	2.9	4.1
Hospitalized pneumonia	1755.4	639.6	639.6	127.1	47.8	118.9	405.2	1730.1
Non-hospitalized pneumonia	438.8	1901.6	1901.6	377.7	229.8	571.8	1082.4	2760
Otitis media	300	300	300	-	-	-	-	-
**Proportion of IPD cases, %**								
Meningitis	33.27	0	0	33.27	0	33.55	31.82	17.92
Bacteremia								
**Fatality rate, %**								
Meningitis	10	10	10	10	6.9	7.3	11.2	14.11
Bacteremia	3	3	3	3	6.9	7.3	11.2	14.11
Hospitalized pneumonia	0.29	0.29	0.29	0.51	1.7	3.96	5.3	7.43
**Other Inputs**								
**Vaccine uptake, %**								
PCV13/15/20	96.1							
**Incoming birth cohort**								
No. of persons	43,652 – 48,047							

South Korea [17]	Age group							
	**<12 mo**	**12–23 mo**	**24–59 mo**	**5–17 yrs**	**18–34 yrs**	**35–49 yrs**	**50–64 yrs**	**≥65 yrs**
**Population size**								
No. of persons	244,250	264,788	277,529	307,975	10,698,816	11,371,371	12,830,101	9,259,557
**Serotype coverage, %**								

PCV13	13.9	13.9	13.9	25	19.1	19.1	30.9	34.3
PCV15	18.5	18.5	18.5	33.3	27	27	39.8	40
PCV20	62.7	62.7	62.7	49.9	55.4	55.4	53.7	59.8
**Disease incidence per 100,000 individuals**								
IPD	0.8	0.8	0.8	0.1	0	0.1	0.1	0.7
Hospitalized pneumonia	5136	5136	5136	1283	345	370	365	675
Non-hospitalized pneumonia	25889	25889	25889	6465	1740	1866	1840	3404
Otitis media	28089	28089	28089	-	-	-	-	-
**Proportion of IPD cases, %**								
Meningitis	39.4	39.4	39.4	56.4	51.1	25.9	33.3	10.4
Bacteremia								
**Fatality rate, %**								
Meningitis	15.6	15.6	11.6	6.4	18.4	18.4	26.9	40.7
Bacteremia	5.6	5.6	5.6	5.6	18.4	18.4	26.9	40.7
Hospitalized pneumonia	0.1	0.1	0.1	0.1	7	7	7	7
**Other Inputs**								
**Vaccine uptake, %**								
PCV13/15/20	97							
**Incoming birth cohort**								
No. of persons	167,071 – 238,359							

**Spain [18]**	**Age group**							
	**<12 mo**	**12–23 mo**	**24–59 mo**	**5–17 yrs**	**18–34 yrs**	**35–49 yrs**	**50–64 yrs**	**≥65 yrs**
**Population size**								
No. of persons	328,532	337,089	1,085,424	6,278,154	9,120,969	10,806,843	10,744,195	9,911,902
**Serotype coverage, %**								
PCV13	22.4	22.4	35.9	32.7	28	28	28	2.8
PCV15	33.5	33.5	46.6	38.8	34.3	34.3	34.3	2.8
PCV20	58.2	58.2	57.3	72.4	75.4	75.4	75.4	34.6
**Disease incidence per 100,000 individuals**								
IPD	27.1	27.1	11	11	4.3	4.3	4.3	15.6

Hospitalized pneumonia	556	556	293	54.5	73.4	73.4	229	1437.7
Non-hospitalized pneumonia	1409.3	1409.3	1409.3	221.1	104	201.6	343.4	875
Otitis media	24,290	24,290	24,290	17,294.3	-	-	-	-
**Proportion of IPD cases, %**								
Meningitis	17	11.36	11.36	11.36	1.41	1.59	1.81	0.64
Bacteremia								
**Fatality rate, %**								
Meningitis	3.23	2.27	2.27	1.67	4.28	4.28	3.33	16.86
Bacteremia	4.62	8.24	8.24	7.6	3.85	3.85	5.27	14.91
Hospitalized pneumonia	0.3	0.3	0.2	0.85	0.48	0.48	1.49	10.43
**Other Inputs**								
**Vaccine uptake, %**								
PCV13/15/20	92.26							
**Incoming birth cohort**								
No. of persons	333,112 – 368,640							

**Sweden [19]**	**Age group**							
	**<12 mo**	**12–23 mo**	**24–59 mo**	**5–17 yrs**	**18–34 yrs**	**35–49 yrs**	**50–64 yrs**	**≥65 yrs**
**Population size**								
No. of persons	105,186	116,123	355,058	1,618,418	2,270,102	1,995,237	1,914,295	2,144,450
**Serotype coverage, %**								
PCV13	18	42	42	35.4	35.4	35.4	35.4	29.7
PCV15	27	57	57	45.8	45.8	45.8	45.8	37.9
PCV20	45	72	72	77.9	77.9	77.9	77.9	57.3
**Disease incidence per 100,000 individuals**								
IPD	5.6	5.6	5.6	5.9	5.9	5.9	5.9	41.5
Hospitalized pneumonia	417	417	201	41	56	119.9	157	1145.3
Non-hospitalized pneumonia	2024.2	2024.2	975.7	199	271.8	582	762.1	1137
Otitis media	17,100	17,100	17,100	4,490	-	-	-	-
**Proportion of IPD cases, %**								
Meningitis	8	8	8	8	8	8	8	8

Bacteremia	92	92	92	92	92	92	92	92
**Fatality rate, %**								
Meningitis	6.6	5.8	5.8	16.8	16.8	16.8	16.8	21.8
Bacteremia	6.6	5.8	5.8	16.8	16.8	16.8	16.8	21.8
Hospitalized pneumonia	0.15	0.09	0.09	0.9	4.42	4.42	11.94	18.67
**Other Inputs**								
**Vaccine uptake, %**								
PCV13/15/20	97.6							
**Incoming birth cohort**								
No. of persons	102,900 – 108,100							

**Taiwan [20]**	**Age group**							
	**<12 mo**	**12–23 mo**	**24–59 mo**	**5–17 yrs**	**18–34 yrs**	**35–49 yrs**	**50–64 yrs**	**≥65 yrs**
**Population size**								
No. of persons	130,289	140,812	533,955	2,640,255	4,866,565	5,544,127	5,297,454	4,296,985
**Serotype coverage, %**								
PCV13	25	14.2	24.2	27.2	42.7	44.8	40.7	42.5
PCV15	25	14.2	24.2	27.2	42.7	44.8	41.2	43.1
PCV20	25	27.5	33.7	49.6	50.1	53.7	50.6	50.1
**Disease incidence per 100,000 individuals**								
IPD	0.8	0.7	1.7	0.1	0.2	0.3	1.3	2.4
Hospitalized pneumonia	31.1	1242.5	1580.3	355.7	107.5	272.3	788.9	5456.2
Non-hospitalized pneumonia	115.1	6993.6	11,006.7	2803.2	928.7	1629.2	3021.8	11,956
Otitis media	8966.3	11749.3	11764.1	2763.4	-	-	-	-
**Proportion of IPD cases, %**								
Meningitis	0	0	12.5	0	0	0	1.8	0.79
Bacteremia								
**Fatality rate, %**								
Meningitis	2.4	2.4	2.4	2.4	13.4	13.4	13.4	13.4
Bacteremia	2.4	2.4	2.4	2.4	13.4	13.4	13.4	13.4
Hospitalized pneumonia	0.4	0.1	0.1	0.74	2.17	6.34	7	8.71

Other Inputs	
**Vaccine uptake, %**	
PCV13/15/20	96.8
**Incoming birth cohort**	
No. of persons	139,521 – 159,063

**USA [21]**	**Age group**							
	**<12 mo**	**12–23 mo**	**24–59 mo**	**5–17 yrs**	**18–34 yrs**	**35–49 yrs**	**50–64 yrs**	**≥65 yrs**
**Population size**								
No. of persons	3,783,050	3,829,599	11,964,032	53,462,467	76,159,527	62,056,895	62,925,688	54,058,264
**Serotype coverage, %**								
PCV13	20.5	20.5	24.5	25.8	28.3	28.3	25.4	24.9
PCV15	39.8	39.8	38.8	37.9	39.7	39.7	38.8	40.3
PCV20	55.4	55.4	49	57.6	57.9	57.9	55.7	52.9
**Disease incidence per 100,000 individuals**								
IPD	13.7	10.4	4.3	1.4	2.3	6.9	15.6	23.7
Hospitalized pneumonia	684	485	235	94	122.8	122.8	475.9	1477.2
Non-hospitalized pneumonia	2007	3774.5	3268	1245.4	622.9	622.9	1104.3	2864
Otitis media	64,770	62,218	38,974	11,765	-	-	-	-
**Proportion of IPD cases, %**								
Meningitis	7	7	7	7	7	7	7	7
Bacteremia	93	93	93	93	93	93	93	93
**Fatality rate, %**								
Meningitis	7.01	5.26	3.45	4.62	4.88	7.91	11.1	14.17
Bacteremia	7.01	5.26	3.45	4.62	4.88	7.91	11.1	14.17
Hospitalized pneumonia	1.3	0.53	0.4	1.34	1.4	1.4	3.8	7.97
**Other Inputs**								
**Vaccine uptake, %**								
PCV13/15/20	89.7							
**Incoming birth cohort**								
No. of persons	3,809,354 – 4,007,572							

References

[1] Rey-Ares, L., et al., *Cost-effectiveness analysis of the pediatric 20-valent pneumococcal conjugate vaccine compared with lower-valent alternatives in Argentina.* Vaccine, 2024. **42**(23): p. 126043.

[2] Struwig, A., Ta, A., Thorat, A., Ilic, A., Warren, S., *Evaluating the Implementation of the 20-Valent Pneumococcal Conjugate Vaccine for Paediatric Immunization in Australia.* SSRN, 2025.

[3] Mignon, A., Ta, A., De Sutter, E., Taelman, A., Ilic, A., Perdrizet., J., *Public health impact of implementing the 20-valent pneumococcal conjugate vaccine for routine paediatric vaccination in belgium*, in *European Society for Paediatric Infectious Diseases (ESPID)*. 2025: Bucharest, Romania.

[4] Lytle, D., et al., *Cost-effectiveness analysis of PCV20 to prevent pneumococcal disease in the Canadian pediatric population.* Hum Vaccin Immunother, 2023. **19**(2): p. 2257426.

[5] Bolanos, R., Falconi, J., Ta, A., Huang, L., *Cost-effectiveness analysis of 20-valent pneumococcal conjugate vaccination versus standard of care (13-valent pneumococcal conjugate vaccination) and 15-valent pneumococcal conjugate vaccination in chilean children*, in *European Society for Paediatric Infectious Diseases (ESPID)*. 2025: Bucharest, Romania.

[6] Fievez, S., et al., *A Cost-Effectiveness Analysis of the Switch to 20-Valent Pneumococcal Conjugate Vaccine from Lower-Valent Pneumococcal Conjugate Vaccines in the French Pediatric Population.* Infect Dis Ther, 2025. **14**(9): p. 2171-2187.

[7] Ta, A., et al., *Cost-effectiveness of PCV20 to Prevent Pneumococcal Disease in the Pediatric Population: A German Societal Perspective Analysis.* Infect Dis Ther, 2024. **13**(6): p. 1333-1358.

[8] Tzanetakos, C., et al., *Cost-effectiveness analysis of 20-valent pneumococcal conjugate vaccine (PCV20) to prevent pneumococcal disease in the Greek pediatric population.* Expert Rev Vaccines, 2025. **24**(1): p. 486-498.

[9] Basile, M., et al., *An Italian cost-utility analysis of 20-valent pneumococcal conjugate vaccine for routine vaccination in infants.* J Med Econ, 2025. **28**(1): p. 674-687.

[10] Shinjoh, M., et al., *Cost-effectiveness analysis of 20-valent pneumococcal conjugate vaccine for routine pediatric vaccination programs in Japan.* Expert Rev Vaccines, 2024. **23**(1): p. 485-497.

[11] Ta, A., Loh E., Yogarajah, P., Teh, N., Peixoto, T., Huang, L., *Analyzing the cost-effectiveness of introducing the 20-valent pneumococcal conjugate vaccine into malaysia's national pediatric immunization program*, in *European Society for Paediatric Infectious Diseases (ESPID)*. 2025: Bucharest, Romania.

[12] Huerta, J.L., et al., *PCV20 for the prevention of invasive pneumococcal disease in the Mexican pediatric population: A cost-effectiveness analysis.* Hum Vaccin Immunother, 2025. **21**(1): p. 2475594.

[13] Mota C, T.A., Vinand E, Teixeira R, Ilic A, Perdrizet J., *Cost-Effectiveness of Switching From PCV13 to PCV20 in the Portuguese Childhood National Immunization Program*, in *The Professional Society for Health Economics and Outcomes Research Europe (ISPOR EU)*. 2024: Barcelona, Spain.

[14] Ta, A., Cheong, E., Vinand, E., Huang, L., *Cost-effectiveness analysis of 20-valent pneumococcal conjugate vaccine implementation into singapore's paediatric national immunisation programme*, in *European Society for Paediatric Infectious Diseases (ESPID)*. 2025: Bucharest, Romania.

[15] Ta, A., Cheong, E., Vinand, E., Huang, L., *Cost-effectiveness analysis of 20-valent pneumococcal conjugate vaccine implementation into singapore's paediatric national immunisation programme*, in *European Society for Paediatric Infectious Diseases (ESPID)*. 2025: Bucharest, Romania.

[16] Wannaadisai W, K.L., Šebo P, Hroncova D, Perdrizet J., *Cost-effectiveness of a 20-valent pediatric pneumococcal conjugate vaccine compared with 13-valent and 15-valent pneumococcal conjugate vaccines in Slovakia.* , in *European Society for Paediatric Infectious Diseases (ESPID)*. 2024: Copenhagen, Denmark

[17] Kang, D.W., et al., *Cost-effectiveness analysis of the 20-valent pneumococcal conjugate vaccine for the pediatric population in South Korea.* Vaccine, 2024. **42**(22): p. 126000.

[18] García, F., et al., *Cost-effectiveness analysis of 20-valent anti-pneumococcal vaccination in the Spanish pediatric population.* Expert Rev Pharmacoecon Outcomes Res, 2025: p. 1-13.

[19] Fridh, A.C., et al., *An economic evaluation of pneumococcal conjugate vaccines, PCV20 versus PCV15, for the prevention of pneumococcal disease in the Swedish pediatric population.* Hum Vaccin Immunother, 2024. **20**(1): p. 2400751.

[20] Yang, Y., Wang, Y., Ta, A., Peixoto, T., Huang, L., *Cost-effectiveness of implementing the 20-valent pneumococcal conjugate vaccine, under different schedules, in the pediatric national immunization program in taiwan*, in *European Society for Paediatric Infectious Diseases (ESPID)*. 2025: Bucharest, Romania.

[21] Rozenbaum, M.H., et al., *Cost-effectiveness of 20-valent pneumococcal conjugate vaccine in US infants.* Vaccine, 2024. **42**(3): p. 573-582.
